# The impact of vitamin D food fortification and health outcomes in children: a systematic review and meta-regression

**DOI:** 10.1186/s13643-020-01360-3

**Published:** 2020-06-16

**Authors:** Reem Al Khalifah, Rawan Alsheikh, Yossef Alnasser, Rana Alsheikh, Nora Alhelali, Ammar Naji, Nouf Al Backer

**Affiliations:** 1 Division of Pediatric Endocrinology, Pediatric Department, College of Medicine, King Saud University, Riyadh, Saudi Arabia; 2 College of Medicine, King Saud University, Riyadh, Saudi Arabia; 3 Pediatric Department, King Saud University, Riyadh, Saudi Arabia; 4 Pediatric Department, BC Children’s Hospital, Vancouver, BC Canada; 5 Division of Developmental-Behavioral Pediatric, Pediatric Department, College of Medicine, King Saud University, Riyadh, Saudi Arabia

**Keywords:** Vitamin D, Food fortification, Pediatric, Meta-analysis

## Abstract

**Objective:**

Vitamin D (vitD) deficiency is a global childhood health problem. Food fortification is a promising strategy to curb vitD deficiency. We aimed to assess the effectiveness of utilizing vitD fortification in staple foods to improve 25hydroxyvitamin D (25(OH)D) concentration and to reduce the prevalence of vitD deficiency among healthy children.

**Methods:**

We conducted a systematic review and meta-analysis of randomized controlled trials (RCTs) evaluating the use of vitD fortified food products compared to no fortification among healthy children aged 1–18 years old. We searched Medline, Embase, Global Health, and Cochrane (CENTRAL) databases from database inception until May 2019. Independently, six reviewers in pairs screened titles and abstracts, assessed the full text for eligibility, and performed data extraction and quality assessment. The primary outcome is the impact of fortification on 25(OH)D concentration. The secondary outcomes included the impact of fortification on the prevalence of vitD deficiency, school performance, cognitive function, school absences, infection rate, hospital admission length, and compliance with fortified food product consumption.

**Results:**

We identified 2229 articles. After assessing eligibility, 20 RCTs met the inclusion criteria. The eligible RCTs assessed the fortification of milk, cereal, juice, bread, yogurt, and cheese compared with no fortification. All RCTs, except for three, had a low risk of bias. Food fortification improved 25(OH)D concentration by a mean difference (MD) of 15.51 nmol/L (95% confidence interval (CI) 6.28, 24.74; *I*^2^ = 99%), which resulted in a mean increase of 3 nmol/l for every 100 IU of vitD, when adjusted for baseline 25(OH)D concentration and country latitude. Additionally, the prevalence of vitD deficiency decreased by a risk ratio of 0.53 (95% CI 0.41, 0.69; *I*^2^ = 95%), and cognitive function improved by a MD of 1.22 intelligence quotient (IQ) points (95% CI 0.65, 1.79; *I*^2^ = 0%). The overall evidence quality was high.

**Conclusion:**

VitD food fortification is an effective way to improve 25(OH)D concentration, prevent vitD deficiency, and improve IQ levels.

**Systematic review registration:**

PROSPERO CRD42017057631

## Introduction

Micronutrient malnutrition or “hidden hunger” is a global health problem that affects more than 2 billion people worldwide [1, 2]. Vitamin D (vitD) deficiency is the most frequent micronutrient deficiency globally [3]. The prevalence of vitD deficiency is high among children worldwide. Although estimates vary, vitD deficiency is thought to affect more than 80% of children in developed countries even in countries with ample sunrays [4–7]. The risk for vitD deficiency is higher for pregnant women, children, the elderly, and individuals with dark skin, limited exposure to sunlight, and those living at higher latitudes [4]. VitD plays a critical role in preventing vitD deficiency rickets and maintaining optimal bone health, muscle strength, and immune function [8–13]. Furthermore, recent studies have suggested that vitD has anti-inflammatory and antioxidant properties in controlling asthma, eczema, upper respiratory tract infections (URTI), type 1 diabetes mellitus, and cancer prevention [4, 14–17].

One of the major obstacles contributing to vitD deficiency is the lack of foods naturally rich in vitD. In addition, mass supplementation (Table 1) is less efficacious compared with food fortification because of its higher cost, and insignificant advertisements make mass supplementation difficult to implement or sustain worldwide even among high-risk groups such as premature infants [18–20]. Furthermore, the mild symptoms and subtle signs of vitD deficiency might discourage children and adolescents from taking daily supplements for a long period of time [21]. Even if they agree to take supplements, they may have low compliance rates.

**Table 1:** Definitions

Fortification: is the practice of deliberately increasing the content of an essential micronutrient, i.e. vitamins and minerals (including trace elements) in a food, to improve the nutritional quality of the food supply and provide a public health benefit with minimal risk to health.	
Mass fortification: refers to the addition of micronutrients to foods commonly consumed by the general public, such as cereals and milk.	
Supplementation: refers to the intake of a specific micronutrient in the form of a supplement.	
Bolus therapy: refers to the intake of a single, large dose of vitD as oral or as an injectable formulation.	

Inadequate vitD intake is a public health problem that can be potentially be eliminated by mandating passive interventions such as vitD food fortification. Food fortification to prevent micronutrient deficiency represents a scalable intervention that is suitable for both developed and developing countries, and it might be easier to implement and sustain among children and adolescents than supplementation [22]. Typically, food is fortified with either vitD2 (ergocalciferol) or vitD3 (cholecalciferol). VitD can be added to food during the manufacturing process or simply by adding/sprinkling it on the food immediately before consumption. Both vitD forms have similar biological activities and sensitivities to oxygen and moisture. VitD is heat stable which enables more food fortification choices [23]. However, it is critical to select a type of food that is appealing to children and culturally accepted to ensure sustainability of intake.

A recent pediatric meta-analysis of nine randomized controlled trials (RCTs) showed greater advantages of using food fortification over supplementation and bolus therapy to improve 25 hydroxyvitamin D (25(OH)D) concentration [20]. However, that meta-analysis failed to include some of the existing RCTs in the literature, and other RCTs that have since been published, mandating an evidence update. In addition, no meta-analysis synthesized the evidence to inform policymakers about the potential impact of fortifying different food products and the impact on health outcomes. Therefore, we aimed to determine the effectiveness of the vitD fortification of staple foods compared with no fortification on 25(OH)D concentration, vitD deficiency prevalence, school performance, cognitive function, school absences, infection rate, and hospital admission length in healthy children aged less than 18 years old. In addition, we determined the effects of fortifying different food products and when those strategies are used in high- or low-income countries.

## Methods

This systematic review and meta-analysis was registered with PROSPERO (CRD42017057631). The report of the systematic review follows the PRISMA recommendations.

### Types of studies

Eligible studies included parallel RCTs, the first period of crossover RCTs, and cluster RCTs.

### Types of participants

We included studies that recruited healthy children aged 1–18 years old and excluded studies that included premature infants or children with chronic diseases such as kidney, liver, or heart failure; malabsorption syndromes (because they have different requirements compared with healthy children); or children taking drugs that affect vitD metabolism (anticonvulsants, steroids, anti-fungal medications).

### Intervention and comparison

We included studies designed to evaluate the effects of vitD fortification as a single micronutrient or as part of a multivitamin fortification of any dose and added to any food product compared with no food fortification or placebo for any period of time.

### Outcomes

The primary outcome was the impact of fortification on 25(OH)D concentration. The secondary outcomes included the impact of fortification on the prevalence of vitD deficiency, school performance, cognitive function, school absences, infection rate, hospital admission length when children required admission because of acute illness acquired during the trial, and compliance with the intervention. A sufficient 25(OH)D concentration was defined as > 75 nmol/L [3, 24, 25].

### Data collection synthesis and analysis

#### Search strategy

We performed literature searches through of Medline, Embase, Global Health using the OVID platform, and Cochrane Central Register of Controlled Trials (CENTRAL) from the database inception date until May 9, 2019. We also checked the reference lists of the included trials for other eligible trials. The search was not limited to a region or to a language. The search terms included combinations of subject headings and keywords pertaining to children, vitD, and fortification (Additional file 1). We used the RCT filter created by McMaster University for Ovid Embase and the Cochrane Library for Ovid Medline [26, 27].

#### Study selection

We used the online systematic review management program Covidence (www.covidence.org) for the process of study selection and data extraction process. Six reviewers in pairs independently screened titles and abstracts based on the inclusion and exclusion criteria. Then, they assessed the full texts of those abstracts for eligibility. At every study selection stage, each reviewer completed a pilot test independently. If there were disagreements between two reviewers at any stage, the principal investigator resolved it after discussion with the other reviewers. Additionally, we checked for multiple publications of the same trial by checking the trial registration number and trial methods.

#### Data extraction

Six reviewers in pairs independently performed the data extraction and risk of bias (RoB) assessment independently. Data were extracted for the country, type of setting, inclusion criteria, exclusion criteria, study design, age, body mass index (BMI), type of food fortified, type of control, vitD dose per day, calcium dose, duration of intervention, number of children randomized, number of children lost to follow-up, reasons for loss to follow-up, vitD level at baseline, the scale for measuring school performance, school absence, cognitive function, type of reported infections, length of hospital admission, and compliance with fortification. We extracted the number of participants in each treatment arm, the mean and standard deviation (SD) or median and range for continuous data, and the number of events for dichotomous outcomes. For cluster trials, we extracted the cluster-adjusted treatment effects, standard errors, intraclass correlation coefficients (ICCs), number of clusters, and cluster unit. All reviewers attended a pilot testing session on data extraction and methodological assessment session and performed data extraction independently.

#### Risk of bias and evidence quality

We used the Cochrane tool to evaluate the RoB in the included RCTs. The RoB tool assesses randomization sequence generation, concealment of allocation, blinding of participants, personnel and outcome assessors, completeness of follow-up, selective outcome reporting, and presence of other biases. Additionally, in the cluster RCTs, we assessed the presence of recruitment bias, baseline imbalance, loss of clusters, and incorrect statistical analysis. We assigned a judgment of high, low, and unclear RoB according to the Cochrane handbook methods [27]. Additionally, we assessed the evidence quality according to the guidance of the Grading of Recommendations Assessment, Development and Evaluation Working Group (GRADE) [28–32]. Following the GRADE approach, the overall confidence starts high and can be downgraded to moderate, low, or very low. We downgraded the evidence quality based on five elements: RoB, inconsistency, indirectness, imprecision, and publication bias, and upgraded the evidence quality when a large treatment effect was present.

### Statistical analysis

We analyzed the effect estimates of values post-treatment. We reported the mean difference (MD) and 95% confidence interval (CI) for continuous data, and the risk ratio (RR) and calculated the number needed to treat (NNT) for dichotomous outcomes (NNT = 1/absolute risk reduction). We calculated the inflated standard error of the mean (SEM) when the trials did not adjust for a clustering effect using the formula recommended form by the Cochrane Collaboration Handbook. The ICC is used to calculate the inflated SEM for cluster RCTs to account for the cluster design effect. When the ICC was not reported by a trial, we chose a value of 0.068, which corresponds to the 95th percentile for adjusted ICCs for individual or cluster characteristics reported in the literature [33–35]. This value was chosen because there was no ICC value reported from any of the included cluster RCTs. Furthermore, we combined study arms for studies that reported the fortification of vitD for more than one arm but with the same food vehicle. The data that were reported as median were converted to mean and SD using the formula recommended form by the Cochrane Collaboration Handbook [27, 36]. Trial data presented as the geometric mean and interquartile range were summarized narratively [27, 37]. Data were pooled using the random effects model. We assessed heterogeneity through the visual inspection of the forest plots, chi-square test, and *I*^2^ statistics to quantify heterogeneity: an *I*^2^ > 50% is considered substantial heterogeneity. We hypothesized that the following variables could explain the observed heterogeneity between studies, and we performed the subgroup analyses accordingly: type of food vehicle, age groups (2–5 years, 5–12 years, and > 12 years) for the studies that used fortified milk (because milk is considered the most common liquid consumed by children worldwide after water), country income, difference in the methodological quality of the studies (high vs. low RoB), and RCT type (cluster vs. parallel). We performed the statistical analysis by the Cochrane Collaboration Review Manager (RevMan version 5.3) [38] and R software using the “meta” package to calculate the MD from the standardized mean difference (SMD) data, because reporting the MD using a familiar scale aids with clinical interpretation and facilitates knowledge translation [39].

We estimated the relationship between the consumption of vitD from fortified foods and the serum 25 (OH)D concentration using a multivariate meta-regression model that controlled for baseline vitD levels and country latitude. Previous adult and pediatric meta-analyses showed a dose-dependent increase in 25(OH)D concentration by 3 nmol/L per 100 IU vitD intake and increased 25(OH)D concentration when the baseline 25(OH)D concentration was < 50 nmol/L, and the country latitude was at ≥ 40° N [20, 40]. We performed the analyses using the “meta” and “‘metafor” packages for meta-analyses on R software [39]. To evaluate the regression assumptions, we assessed the variables, the regression coefficients, normality, and interactions of the variables in the univariate model. Checking for the presence of interaction between included variables is essential for model building because if such interaction is not accounted for, the estimates from the regression coefficient and level of significance are biased. The univariate analyses assessed the vitD fortification dose, baseline 25(OH)D concentration, and country latitude.

Finally, we assessed publication bias using a funnel plot and Egger’s test. Funnel plot provides a visual assessment of the size of the trials plotted against the effect size they report [27]. Typically, a judgment of asymmetry in the study’s results with more studies showing a positive result than a negative result leads to the suspicion of publication bias. However, Egger’s test assesses publication bias statistically. This test has a relatively low power to detect publication bias. Therefore, even when the results are not statistically significant, publication bias cannot be completely excluded.

## Results

### Search for studies

**Fig. 1:**
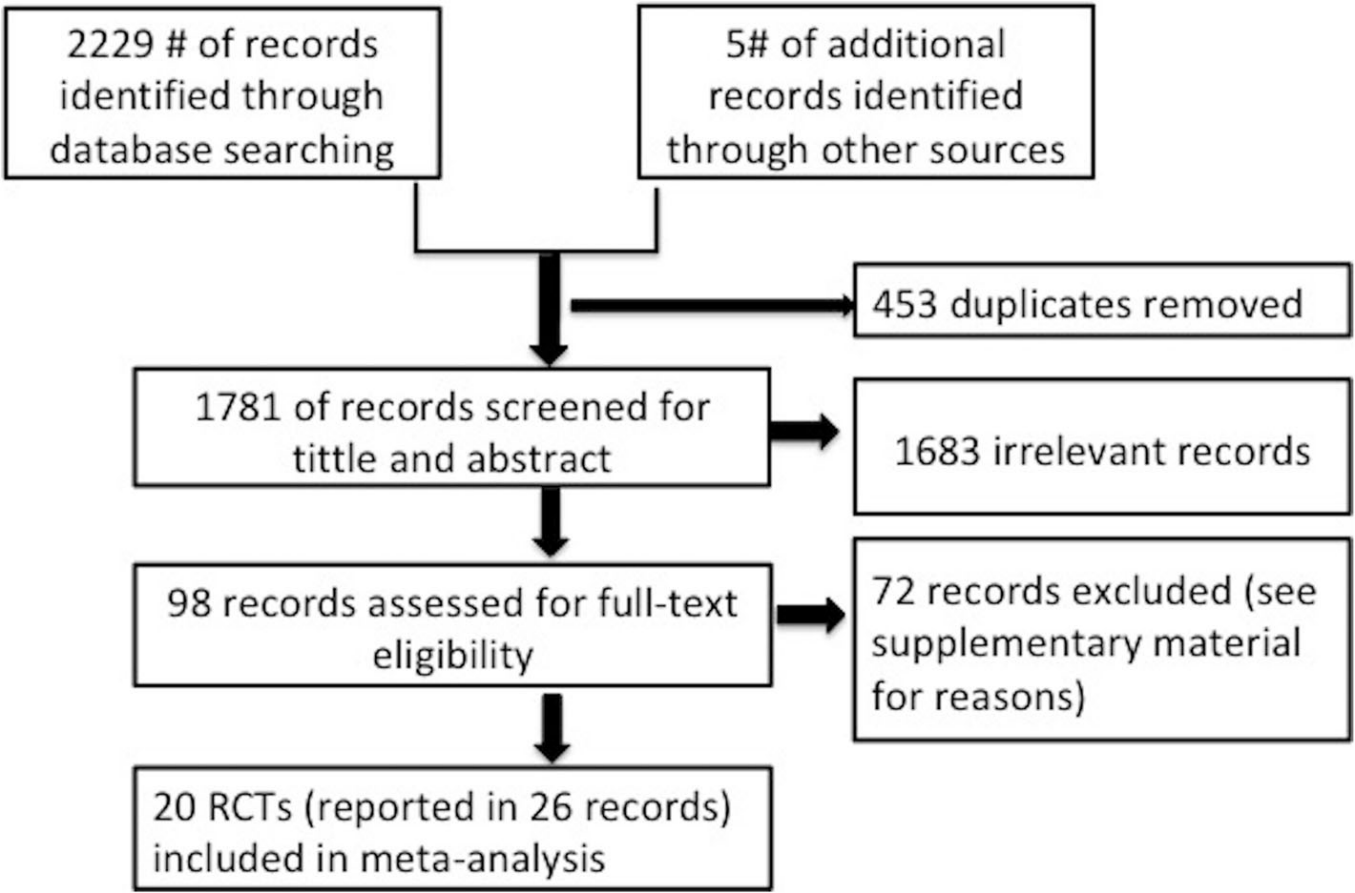
Study flow diagram

We identified 2229 articles from the four databases. After removing duplicates, 1781 articles were screened for the title and abstract eligibility. Subsequently, the full texts of 98 articles were reviewed. We included 20 RCTs (described in 26 papers) that met the eligibility criteria (Fig. 1). The citation of the excluded articles, along with the reason for exclusion, can be found in the supporting information (Additional file 1).

### Study characteristics

We identified 20 RCTs, including 15 parallel RCTs and 5 cluster RCTs, and no crossover trials were identified. The RCTs were conducted in Canada, China, Denmark, Germany, India, Iran, Mongolia, Morocco, New Zealand, Sri Lanka, Sweden, the UK, and the USA. The trials were conducted at 7–56° altitude. The average cluster size, number of clusters, and cluster unit for the cluster trials are reported in Additional file 1. The included children ranged in age from 1.4 to 18 years old. The interventions included the fortification of a single food item such as milk, cereal, juice, and bread and two items of food such as yogurt and cheese or milk and bread. All fortified food products were compared with unfortified food, different food products, or no food. The median intervention duration was 5 months (range 2–24 months) (see Table 2) (Additional file 1) [41–43]. The mean 25(OH)D concentration at baseline was 24.02 nmol/L (95% CI 23.14, 24.91), and the prevalence of vitD deficiency was 46.6%.

**Table 2:** Baseline characteristics of the included studies

Study	Country	County latitude	Country income *	Study Setting	Total randomized patients, ***n***	Lost follow-up, ***n***	Intervention duration, months	Treatment arm	Vit D dose, IU/day	Vit D isoform	Added calcium, mg/day	Age, years mean (SD)	BMI, kg/m^**2**^; mean (SD)	Baseline 25(OH)D concentration, nmol/L	Outcomes
**Akkermans et al. 2017[**41**]**	Western Europe	Not clear	High	Clinical	325	91	5	Milk^**I**^	348**	D3	110	1.7 (0.6)	0.3 (1.0)^**Z**^	69.4 (27.0)	-Vit D concentration -Vit D deficiency -Compliance
								Milk^**C**^	–		127	1.7 (0.6)	0.3 (1.1)^**Z**^	70.2 (26.7)	
**Battiprolu et al. 2006[**51**]**	India	17°	Lower middle	School	328	85	14	Milk^**I**^	400	–	400	10.8 (2.0)	15.2 (1.0)	74.0 (10.0)	-Vit D concentration -School performance -School absences -Compliance
								Milk^**C**^	–		176	10.6 (2.0)	15.4 (1.2)	87.0 (15.0)	
**Benjeddou** et al. **2019[**57**]**	Morocco	32°	Lower middle	School	239	39	9	Milk^**I**^	120	D3	240	7–9	15.4 (10)	53.04 (22.38)	-Vit D concentration -Vit D deficiency
								Milk^**C #**^	60		240				
**Brett et al. 2016[**52**]**	Canada	45°	High	Clinical	77	3	3	Yogurt and cheese^**I**^	400	D3	–	4.9 (2.1)	0.4 (0.6)^**Z**^	59.5 (13.0)	-Vit D concentration -Vit D deficiency -Compliance
								Yogurt and cheese^**I**^	600		–	5.3 (2.0)	0.5 (0.9)^Z^	61.0 (10.6)	
								Yogurt and cheese^**C #**^	140–195**		–	5.0 (1.8)	0.6 (1.0)^**Z**^	58.6 (14.4)	
**Brett 2018[**58**]**	Canada	45°	High	Daycare	51	2	6	Yogurt and cheese^**I**^	300	D3	–	5 (1.8)	0.55 (0.98)^**Z**^	65.3 (12.2)	-Vit D concentration -Vit D deficiency
								Yogurt and cheese^**C #**^	167**		–	5.4 (2.0)	0.81 (0.88)^**Z**^	67.5 (15.1)	
**Du et al. 2004[**49**]**	China	39°	Upper middle	School	757	59	24	Milk^**I**^	133	D3	245	10.1 (0.3)	16.8 (2.6)	20.6 (8.8)	-Vit D concentration -Compliance
								Milk^**C**^	–		245	10.1 (0.4)	17.1 (2.8)	17.7 (8.7)	
								No intervention ^D^	–		–	10 (0.3)	16.8 (2.6)	19.1 (7.4)	
**Economos et al. 2014[**42**]**	USA	42°	High	Clinical	176	34	3	Juice^**I**^	400	-–	1400	8.1 (1.5)	18.5 (4.2)	64.2 (92.8)	-Vit D concentration -Vit D deficiency -Compliance
								Juice+ Vit E+ Vit A^**I**^	400		1400	8.15 (1.4)	18.1 (4.0)	75.6 (27.2)	
								Juice^**C**^	--		1400	7.9 (1.4)	18.5 (4.8)	64.3 (20.9)	
**Graham et al. 2009[**46**]**	New Zealand	37°	High	School	172	-	24	Milk^**I**^	60	D3	480	7.6 (0.9)	–	–	-Vit D concentration -Vit D deficiency
								No milk^**C**^	–		–	7.2 (0.8)	–	–	
**Hettiarachchi et al. 2010[**48**]**	Sri Lanka	7°	Lower middle	Clinical	60	0	9	Cereal^**I**^	100	D3	450	4.0(0.6)	13.6 (1.2)	71.95 (32.3)	-Vit D concentration
								Cereal^**C**^	–		0	4.1 (0.6)	13.6 (0.8)	103.4 (26.4)	
**Houghton et al. 2011[**43**]**	New Zealand	46°	High	Clinical	225	44	5	Milk^**I**^	252	–	132	1.4	–	52.8 (18.4)	-Vit D concentration -Vit D deficiency
								Micronutrient fortified milk ^**I**^	240		102	1.4		48.9 (22.4)	
								Meat^**C**^	–		–	1.4	–	48.8(18.6)	
**Hower et al. 2013[**59**]**	Germany	51°	High	Clinical	92	39	8	Milk^**I**^	392	–	105	3.8 (2.0–6.8)^δ δ^	15.6	53.7(26.5, 112.1) ^δ^	-Vit D concentration -Vit D deficiency -Compliance -Infection rate
								Milk^**C #**^	4.2		116	3.7 (2.0–6.2)^δ δ^	15.4	45.9(25.2, 107.3) ^δ^	
**Khadgawat et al. 2013[**53**]**	India	28°	Lower middle	School	776	63	3	Milk^**I**^	600	D3	–	11.8 (1.1)	18.8 (3.7)	28.5 (13.1)	-Vit D concentration -Vit D deficiency
								Milk^**I**^	1000		–	11.8 (1.1)	18.6 (3.5)	29.8 (14.0)	
								No intervention^**C**^	–		–	11.7 (1.1)	18.9 (3.3)	29.3 (13.0)	
**Kuriyan et al. 2016[**54**]**	India	13°	Lower middle	School	227	3	5	Malt- and cocoa-based milk^**I**^	116	–	492	8.1 (0.8)	–	53.9 (15.7)	-Vit D concentration -Vit D deficiency -Ccognitive function
								Malt- and cocoa-based milk^**C #**^	16		473	8.4 (0.9)	–	54.7 (17)	
**Madsen et al. 2013[**60**]**	Denmark	56°	High	Clinical	321	–	6	Bread and milk^**I**^	408**	D3		4–17	–	72.8	-Vit D concentration -Vit D deficiency -Compliance
								Bread and milk^**C #**^	88			4–17	–	72.8	
**Neyestani et al. 2014[**50**]**	Iran	35.6°	Upper middle	Clinical	146	13	3	Milk^**I**^	100	–	500	9–12	18.3 (0.4)	24.9 (1.4)	-Vit D concentration -Vit D deficiency -Compliance
								Milk^**C**^	–		240		18.3 (0.4)	27.4 (1.9)	
					170	7	3	Orange juice ^**I**^	100	–	500		18.0 (0.3)	24.9 (1.3)	
								Orange juice^**C**^	–		240		17.3 (0.3)	23.8 (1.1)	
**Ohlund et al. 2017[**56**]**	Sweden	63°	High	School and community	206	17	3	Milk^**I**^	400	D3	–	6.3	0.3^**Z**^	56 (52, 60)^$^	-Vit D concentration -Vit D deficiency
								Milk^**I**^	1000			6.3	0.3^**Z**^	58 (53, 62)^$^	
								Milk^**C #**^	80		–	6.3	− 0.1^**Z**^	49 (43, 55)^$^	
**Powers et al. 2016[**55**]**	UK	53°	High	Clinical	78	5	3	Cereal and milk^**I**^	166	–	215	18.8 (1.0)	22.7 (3.1)	42.9 (29.2)	-Vit D concentration -Vit D deficiency -Compliance
								Cereal and milk^**C #**^	8		215	19.0 (1.6)	21.9 (2.5)	39.4 (22.8)	
**Rich-Edwards et al. 2011[**47**]**	Mongolia	48°	Lower middle	School	278	–	2	Mongolian milk^**I**^	300	D3	–	10 (1.0)	17.0 (2.0)	20.0 (10.0)	-Vit D concentration -Vit D deficiency -Compliance -Infection rate
								US milk^**I**^	300		–	10 (1.0)	17.0 (20)	24.9 (12.48)	
								Milk^**C**^	–		–	10 (1.0)	17.0 (20)	20.0 (10.0)	
**Sun 2011[**45**]**	China	43°	Upper middle	School	294	–	12	Milk^**I**^	Not clear	–	–	6–8	–	46.2 (18.9)	-Vit D concentration
								No intervention^**C**^	–		–	6–8	–	45.2 (11.0)	
**Wang et al. 2017[**44**]**	China	34°	Upper middle	School	360	64	6	Milk^**I**^	150	–	100	13.2 (1.0)	21.2 (0.8)	–	-Vit D deficiency -School performance
								Milk^**C**^	–		120	13.4 (0.9)	21.1 (0.7)	–	

*BMI* body mass index, *SD* standard deviation, *Vit D*, vitamin D

*Gross national income (GNI) per capita, World Bank list of economies June 2017

**Estimated average intake of vitamin D per/day

^I^Intervention arm

^C^Control arm

^D^Not included in the analysis

^**z**^z score

^$^95th CI

^δ^Median (IQR)

^δ δ^Median (min, max)

^#^vitD included in the control arms is the naturally occurring vitD in food

### RoB in the included studies

**Fig. 2:**
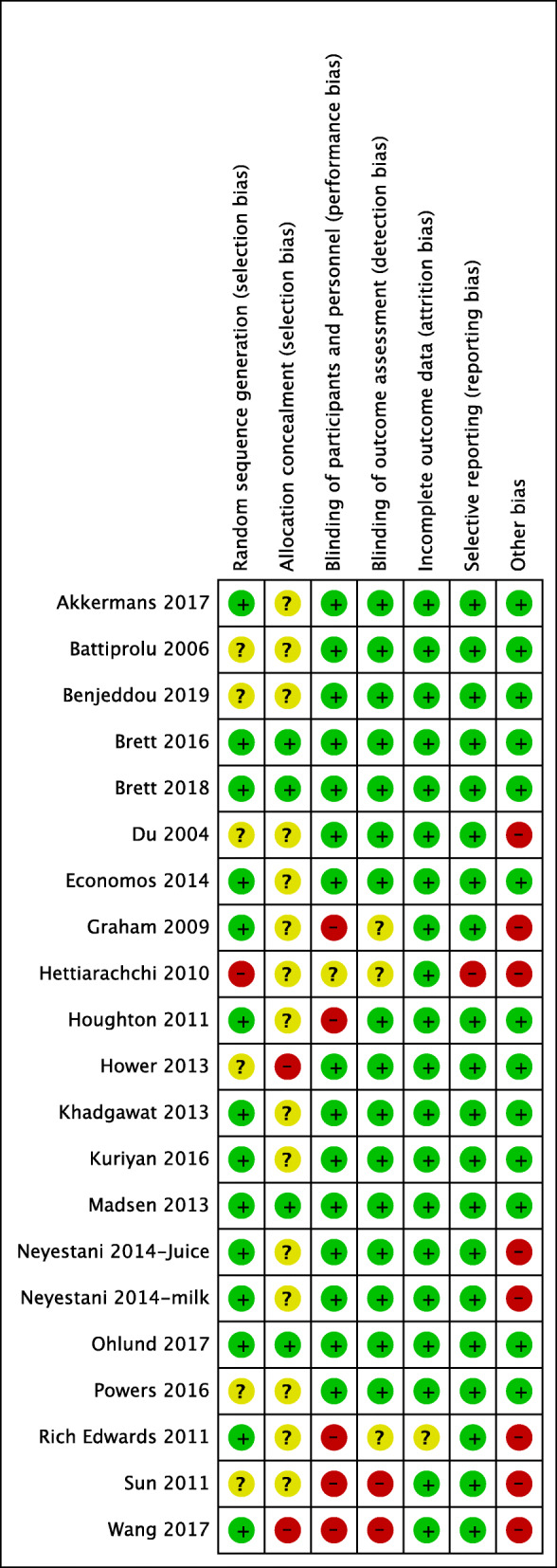
Risk of bias summary

Overall, most of the studies had a low RoB for randomization and an unclear RoB for allocation concealment because none of the RCTs adequately reported on the methods used for allocation concealment. Four studies had a high RoB for blinding because of lack of blinding [43–46]. One study had a moderate RoB for incomplete outcome data because the author did per-protocol analysis that led to the exclusion of 20% of children that were participating in an arm not relevant to the analysis group [47]. For selective outcome reporting, one study was judged to be at high RoB because it seems that the author reported results for children who completed the bone mineral density studies only, and there was no reported loss of follow-up [48]. For the other biases, four out of the five cluster RCTs had a high RoB because they did not account for the cluster design effect [46, 47, 49, 50], and two studies had unclear methods for RCT design [45, 48]. In summary, three studies were determined to have a high RoB (Fig. 2) [44, 45, 48].

### Effect of the intervention

#### 25-Hydroxyvitamin D concentration

**Fig. 3:**
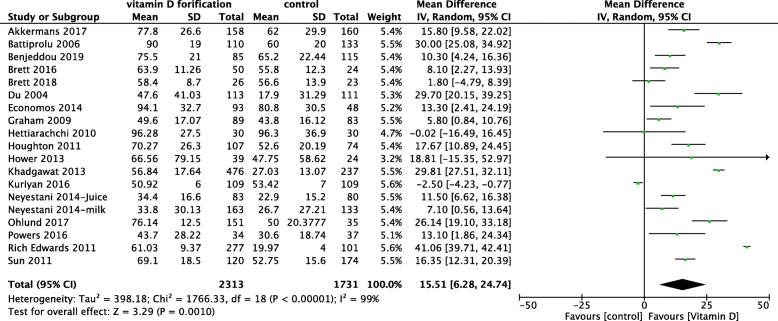
Forest plot of comparison: vitamin D fortification vs control, outcome: 25(OH)D concentration nmol/l

Eighteen RCTs, including 4044 total children, reported the effect of vitD fortification on the mean 25(OH)D concentration [41–43, 45–59]. VitD fortification significantly improved 25(OH)D concentration compared with no fortification by an MD of 15.51 nmol/L (95% CI 6.28, 24.74; *I*^2^ = 99%) (Fig. 3). Madsen et al. reported geometric mean data, which could not be meta-analyzed. At baseline, the 25(OH)D concentration was 72.8 nmol/L for both groups. At the end of the study, despite that the serum 25(OH)D concentration had decreased in both groups, the geometric mean and interquartile range (IQR) of serum 25(OH)D concentration for the vitD fortification group was 67.6 nmol/L (56.2, 79.4) compared to 42.7 nmol/L (30.9, 58.9) for the control group. The difference was statistically significant between the two groups.

##### Subgroup analyses

**Fig. 4:**
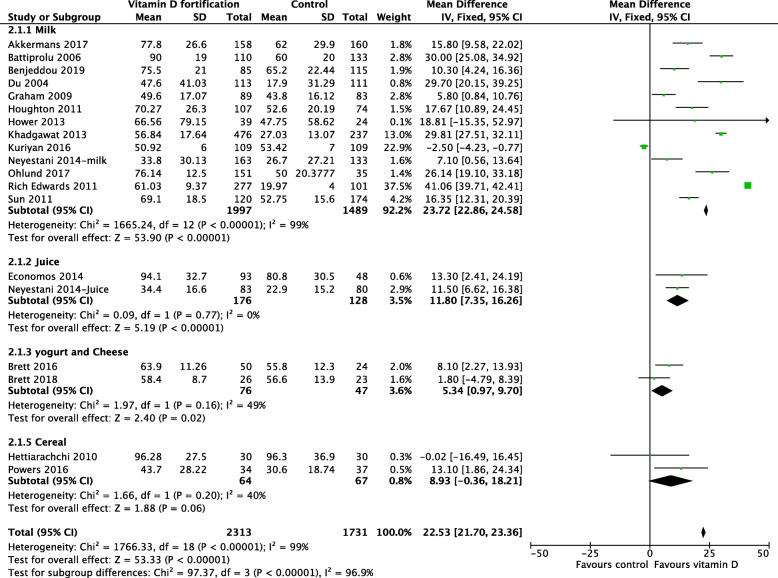
Forest plot subgroup analysis of 25(OH)D concentration based on type of fortified food nmol/l

Given the significant heterogeneity among the included studies, we conducted subgroup analyses based on the food vehicle used, age groups, country income level, the methodological quality of the included studies, and RCT type to explain the heterogeneity. There was a statistically significant difference among the food vehicles used for fortification. Compared with no fortification, fortified milk improved 25(OH)D concentration more than other food vehicles (Fig. 4). Milk increased 25(OH)D concentration by an MD of 23.72 nmol/L (95% CI 22.86, 24.58; *I*^2^ = 99%), juice increased 25(OH)D concentration by an MD of 11.80 nmol/L (95% CI 7.35, 16.26; *I*^2^ = 0%), cereal increased 25(OH)D concentration by an MD of 8.93 nmol/L (95% CI − 0.36, 18.21; *I*^2^ = 40%), and yogurt and cheese increased 25(OH)D concentration by an MD of 5.34 nmol/L (95% CI 0.97, 9.70; *I*^2^ = 49%). Heterogeneity remained substantial among the milk group and was not important for the other subgroups. Although the subgroup analysis is quite possibly underpowered, because the number of studies and participants were sufficient for the milk subgroup only, this subgroup analysis suggests differential effect between fortified food products.

**Fig. 5:**
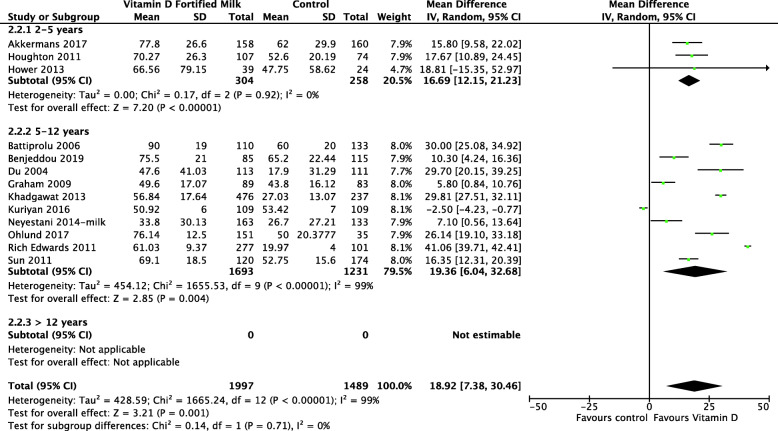
Forest plot subgroup analysis of 25(OH)D concentration based on the children age group among studies that used milk for fortification

**Fig. 6:**
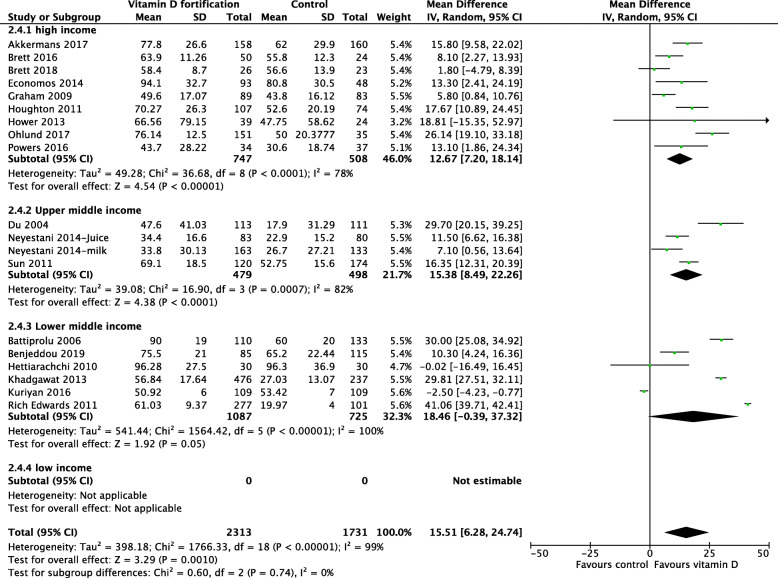
Forest plot subgroup analysis of 25(OH)D concentration based on country level income

**Fig. 7:**
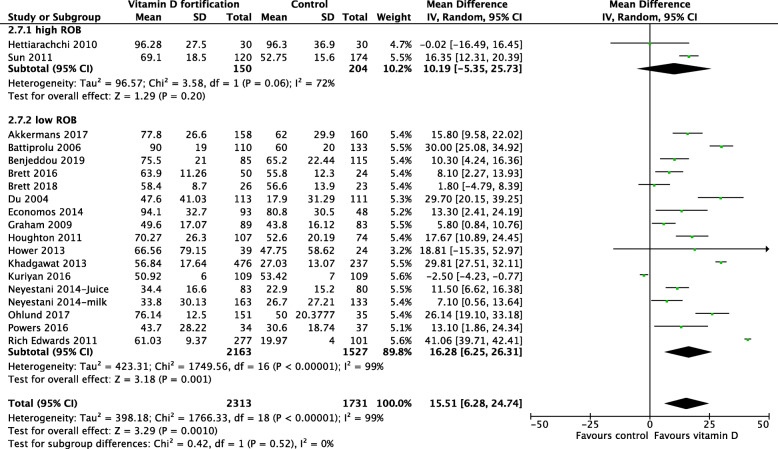
Forest plot subgroup analysis of 25(OH)D concentration based on risk of bias

**Fig. 8:**
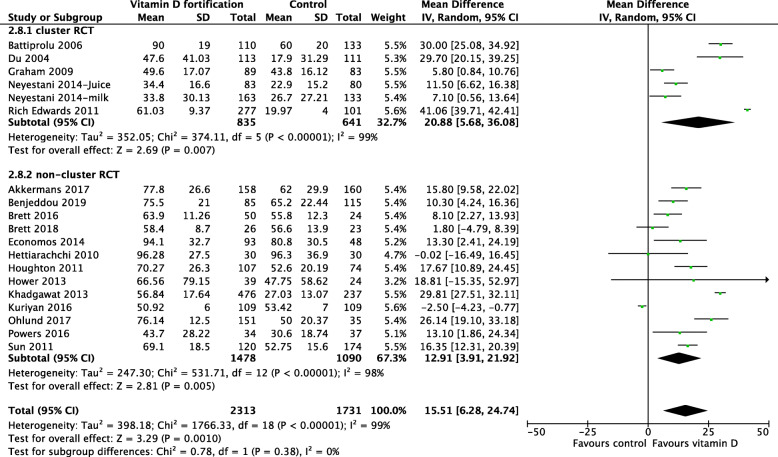
Forest plot subgroup analysis of 25(OH)D concentration based on RCT type

In trials that used milk for fortification, the results showed a clear benefit among preschool and school-aged children. However, school-aged children had higher 25(OH)D concentration compared with pre-school children (Fig. 5). There was no heterogeneity detected in the pre-school group (*I*^2^ = 0%), and the degree of overlap of the point estimates and CIs were homogenous, compared with those in the school-aged children (*I*^2^ = 99%). Subgroup analyses based on country income level (Fig. 6), differences in the methodological quality between studies (Fig. 7), and RCT type (Fig. 8) were not statistically significant.

##### Meta-regression analysis

Table 3 shows the univariate meta-regression analyses. The only variable significant in the univariate model was the vitD dose. There was a significant interaction between latitude and baseline 25(OH)D concentration. The multivariate analysis results are shown in Table 4. The mean changes in 25(OH)D concentration per one-unit increase in vitD fortification dose, baseline 25(OH)D concentration, latitude, and baseline 25(OH)D concentration × latitude were 0.03, 1.26, 2.44, − 0.044, respectively. A total of 76.2% of the between-study variance was explained by the model.

**Table 3:** Univariable meta-regression model

Trial level covariate	Estimate	95% CI	*P* value	Tau^2^	*R*^2^	*I*^2^
Dose, IU	0.02	− 0.0, 0.5	0.02 *	115.7	20.5	96.1
Baseline 25(OH)D concentration, nmol/L	− 0.09	− 0.4, 0.2	0.54	142.7	0.0	96.3
Latitude	0.26	− 0.2, 0.7	0.23	142.2	2.1	96.1

*Tau*^*2*^ unexplained between-study variance, *R*^2^ proportion of total between-study variance explained by the model, *I*^2^ between studies variance, *Statistically significant

**Table 4:** Multivariate meta-regression model

Trial level covariate	Coefficient	95% CI	*P* value	Tau^2^	*R*^2^	*I*^2^
**Intercept**	− 71.08	− 128.09, − 14.07	0.01*	37.1	76.2	80.1
**Dose, IU**	0.03	0.01, 0.05	0.00*			
**Baseline 25(OH)D concentration, nmol/L**	1.26	0.31, 2.21	0.00*			
**Latitude**	2.44	1.02, 3.87	0.001*			
**Latitude × baseline 25(OH)D concentration**^ϑ^	− 0.044	− 0.06, − 0.02	0.00*			

*Tau*^*2*^ unexplained between-study variance, *R*^2^ proportion of total between-study variance explained by the model, *I*^2^ between studies variance

^ϑ^Interaction term

*Statistically significant

#### Vitamin D deficiency prevalence

**Fig. 9:**
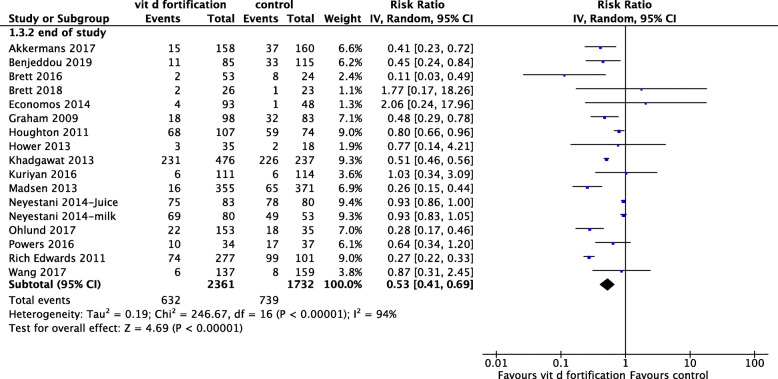
Forest plot of comparison: vitamin D deficiency prevalence

Sixteen RCTs, including 4093 total children, reported a reduction in the prevalence of vitD deficiency after fortification [41–44, 46, 47, 50, 52–60]. Food fortification reduced vitD deficiency by an RR of 0.53 (95% CI 0.41, 0.69; *I*^2^ = 94%) (Fig. 9), indicating that the risk of vitD deficiency is reduced by 0.53-folds compared with no fortification. Additionally, the number needed to treat (NNT) was calculated as 6.3 children to prevent one case of vitD deficiency.

#### School performance and absences

**Fig. 10:**
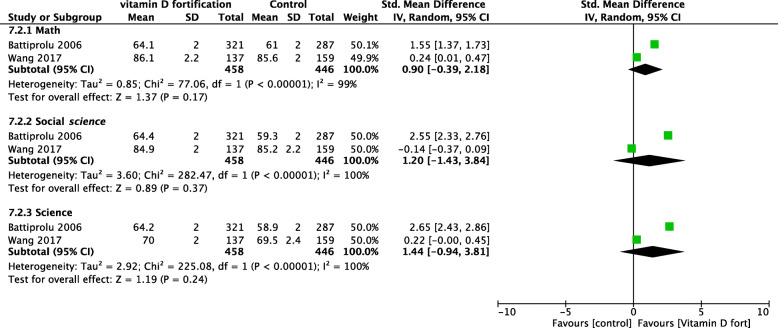
Forest plot of school performance

Two studies reported on school performances in math, science, and social science [44, 54]. Academic performance was measured using age- and gender-standardized end-of-term test scores retrieved from the school administration system. There was no significant difference in the single subjects, including math, social science, and science (Fig. 10). However, the observed heterogeneity was substantial. Battiprolu et al. reported a mean reduction in school absences of 2.4 days over 14 months in the intervention group compared with the control group (95% CI − 2.54, − 2.26) [51].

#### Cognitive function

**Fig. 11:**
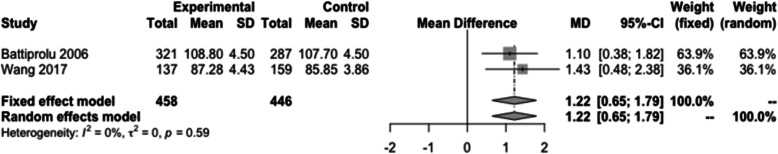
Forest plot of cognitive function (IQ)

A comprehensive cognitive assessment evaluates various areas of cognitive ability, including verbal comprehension, visual–spatial, fluid reasoning, working memory, and processing speed. The gold standard cognitive assessment tool is the Wechsler Scale of Intelligence, formerly known as the intelligence quotient or IQ test. Three studies reported the impact of fortification on cognitive function using different measurement scales [44, 51, 54]. The Motivated Strategies for Learning Questionnaire (MSLQ) was used to assess cognitive and academic performance. It is a self-reported questionnaire that consists of many scales with a total of 56 items. Wang studied student motivation (intrinsic value, self-efficacy, and test anxiety) and learning strategy (strategy use and self-regulation), whereas Battiprolu used the IQ test. VitD fortification significantly improved cognitive function by an MD of 1.22 (95% CI 0.65, 1.79) on the natural scale of IQ (Fig. 11). Table 5 summarizes the results of Kuriyan et al., who used a scale that could not be combined with the other studies.

**Table 5:** Cognitive measures in children at the end of the study by Kuriyan, 2016

Cognitive measures	vitD fortification	Control	*P* value
CCT—no. of correct responses	57.8 ± 4.5	58.4 ± 2.5	0.72
CCT—time taken for correct response (seconds)	88.3 ± 23.4	86.8 ± 27.8	0.57
CTT—trial A no. of correct responses	24.8 ± 0.6	24.9 ± 0.4	0.74
CTT—trial B no. of correct responses	23.4 ± 4.1	24.3 ± 2.0	0.68
Time taken trial A correct response (s)	111.1 ± 45.7	109.7 ± 49.1	0.07
Time taken trial B correct response (s)	194.3 ± 73.1	188.6 ± 77.8	0.71
Word order test—no. of responses	17.2 ± 3.2	17.7 ± 3.7	0.45
Portues maze test—test age (months)	188.1 ± 25.2	191.9 ± 22.5	0.41

Data are shown as the mean ± SD

Trials A and B are subsets of the CTT

Color cancellation test (CCT) (Kapur, 1974): a measure of selective attention/visual scanning and activation and inhibition of a rapid response. It consists of 150 circles in five different colors, i.e., red, blue, yellow, black, and gray. The participants are required to cancel only the yellow and red circles as fast as they can. The time taken in seconds to complete the task is the score

Color trails test (CTT): a measure of focused attention. Children aged 5 to 16 years show a steady age progression on this test. It is sensitive enough to reflect frontal lobe damage

Word order test: it evaluates phonological loop component of short-term memory. It is responsible for holding verbal information for short period of time

The Porteus maze test: a non-language test of executive functioning, planning, and inhibition; it is a nonverbal test of mental ability to measure a nonverbal executive functioning, planning, inhibition, patience, and mental alertness in a novel and concrete performance task; it is particularly accurate at differentiating lower levels of cognitive ability

#### Infection rate and hospitalization

Two RCTs reported the infection rate among those healthy children. Edwards et al. reported a lower chest infection rate among the vitD fortified group (MD − 0.35, 95% CI − 0.58, − 0.12) after 2 months of follow-up [47], while Battiprolu et al. reported no events of URTI or diarrhea in either group after 14 months of follow-up [51]. None of the studies reported hospitalization during the study duration.

#### Compliance with fortification du powers

Compliance was defined differently in the studies. Akkermans et al. defined good compliance as consuming > 151 mL of the study product/day for > 80% of the days within the last 28 days of study product intake [41]. The percentage of good compliance was 69.6% among the intervention group vs. 71.9% in the control group. Brett et al. used parental reports of compliance by using a daily calendar check sheet to keep track of how many of the study products their child consumed each day [52]. Overall, the compliance among the control group was yogurt 89% and cheese 88%, and for the group receiving 400 IU of vitD was yogurt 80% and cheese 79%, and for the group receiving 600 IU of vitD was yogurt 89% and cheese 84%. Du et al. defined compliance as adequate milk intake with no more than 4 days of missing drinking milk. Overall, compliance was close to 100% amongst those who completed the study [49]. Hower et al. retrospectively recorded the consumed volumes of study milks during the 3 study visits [59]. The study milk was consumed on an average of 80% of days of the study duration. During the study, 6/46 children in the intervention and 7/35 in the control group discontinued the study because it was not acceptable anymore. Madsen et al. estimated compliance by dividing the number of portions of milk or bread consumed per day other than the products provided in the study by the total number of portions of milk or bread consumed per day as reported in the food frequency questionnaire [60]. Overall, the compliance for the intervention group was milk 84% and bread 93%, and the control group milk 89% and bread 94%. Power et al. reported compliance by estimating the weight of cereal returned at the end of the study. The intervention group consumed more cereal than asked to consume 102 ± 10.3%, compared to consumption of 98 ± 10.2% for the control group [55]. Overall, compliance with the intake of fortified food products compared to non-fortified food products reported to be similar by six studies.

#### Publication bias

**Fig. 12:**
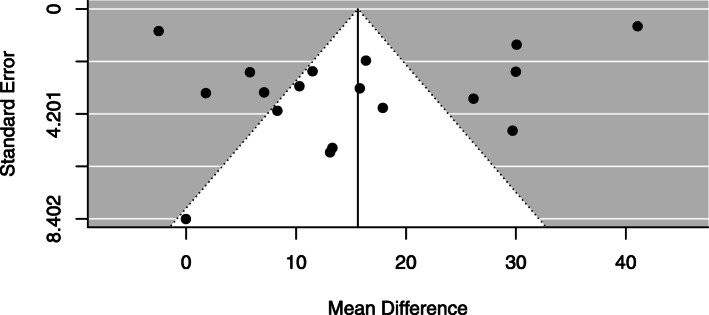
Funnel plot

No publication bias was detected by Egger’s test (*p* value = 0.24). Likewise, the funnel plot was symmetrical for the primary outcome (Fig. 12).

### Certainty of the evidence

**Fig. 13:**
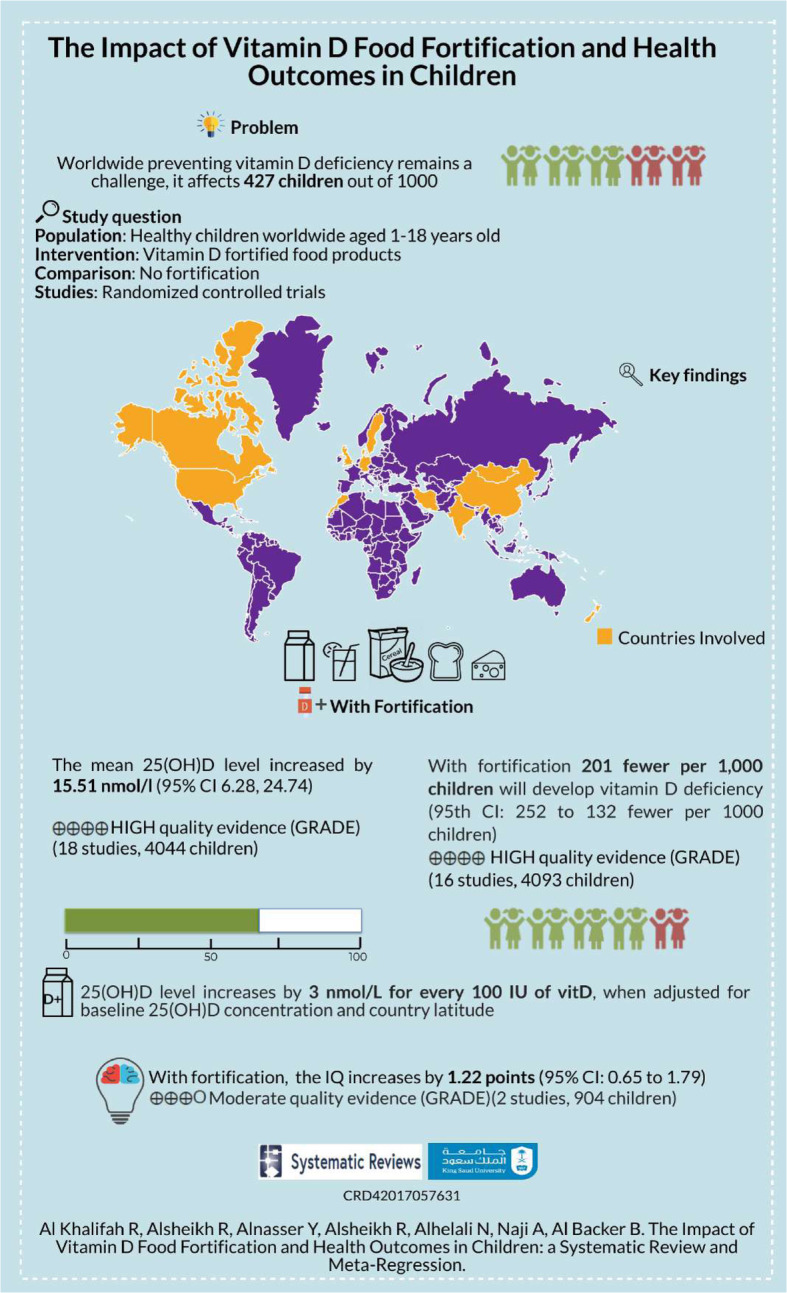
Infographics

The quality of evidence for 25(OH)D concentration and vitD deficiency was high. We rated down the quality of evidence for heterogeneity to “serious” instead of “very serious” because heterogeneity was partially explained in the meta-regression model and in the subgroup analysis of fortified food type. A total of 76.2% of the between-study variance was explained by the model. However, because we observed a large dose-dependent response to vitD food fortification, we upgraded the quality of the evidence to “high” according to the GRADE recommendations (Table 6, Fig. 13).

**Table 6:** GRADE evidence profile for VitD food fortification for preventing vitD deficiency among children

Certainty assessment							Summary of findings				
No. of participants (studies)Follow-up	Risk of bias	Inconsistency	Indirectness	Imprecision	Other considerations	Overall certainty of evidence	Number of children		Relative effect (95% CI)	Anticipated absolute effects	
							No. fortification	Vitamin D fortification		Risk with no fortification	Risk difference with vitamin D fortification
**25(OH)D concentration (follow-up: range 2 to 24 months; assessed with: nmol/L)**											
4044 (18 RCTS)	Not serious	Serious^a, b^	Not serious	Not serious	Strong association^d^	⨁⨁⨁⨁High	1731	2313	–	The mean vitamin D concentration was **44** nmol/L	MD 15.51 **nmol/L higher** (6.28 higher to 24.74 higher)
**Vitamin D deficiency prevalence (follow up: range 2 to 24 months)**											
4093 (16 RCTS)	Not serious	Serious^a^	Not serious	Not serious	Strong association^d^	⨁⨁⨁⨁High	739/1732 (42.7%)*	632/2361 (26.8%)*	**RR 0.53** (0.41 to 0.69)	**427** per 1000	**201 fewer** per **1000** (252 to 132 fewer)
**Cognitive function (follow up: range 6 to 14 months; assessed with: IQ)**											
904 (2 RCTS)	Serious^c^	Not serious	Not serious	Not serious	Strong association^e^	⨁⨁⨁OModerate	446	458	–	-	MD **1.22 IQ points higher** (0.65 higher to 1.79 higher)
**School grade for Math (follow up: range 6 to 14 months)**											
904 (2 RCTs)	Serious^c^	Serious^a^	Not serious	Serious	None	⨁OOOVery LOW	446	458	–	–	SMD **0.90 SD higher** (0.39 lower to 2.18 higher)

The evidence quality table was produced using the online GRADE-Pro-Guidelines Development Tool (www.guidelinesdevelopment.org)

*CI* confidence interval, *MD* mean difference, *RR* risk ratio, *SMD* standardized mean difference

^a^CI not overlapping

^b^Substantial heterogeneity was partially explained by a meta-regression model including vitD dose, latitude, and vitD baseline level. The proportion of total between-study variance explained by the model is 76.2%

^c^Wang et al. (lack of concealment, blinding, and did not use appropriate statistical method for cluster RCT)

^d^Large effect, dose response for every 100 IU vitD, the 25(OH)D concentration increased by 3 nmol/L when controlling for baseline 25(OH)D concentration and latitude

^e^Large effect: increased IQ by 1 point

*Event rate ⨁⨁⨁⨁ high qulity evidence, ⨁⨁⨁O Moderate qulity evidence, ⨁⨁OOO LOW qulity evidence, ⨁OOO Very LOW qulity evidence

## Discussion

The World Health Organization had called for mandatory micronutrient fortification whenever there is a significant public health need or risk for deficiency in a given population [23]. However, many countries worldwide have not implemented voluntary vitD fortification strategies because of limited data on non-skeletal health outcomes and cost-effectiveness [61]. Conversely, mandatory vitD food fortification legislation has been implemented by some of the high-income countries [62]. High-quality evidence from 20 RCTs (*n* = 4044) showed improved 25(OH)D concentration by 15.51 nmol/L and reduced vitD deficiency prevalence by one child for every 6.3 children receiving a vitD fortified food product. Our results are similar to a recent pediatric meta-analysis of nine RCTs that showed a mean increase in 25(OH)D concentration of 6.9 nmol/L (95% CI 3.7, 10.0 nmol/L) with vitD food fortification and to an adult meta-analysis of seven RCTs (*n* = 585) that showed increased 25(OH)D concentration in the fortified group from 14.5 to 34.5 nmol/L [20, 63]. This meta-analysis was updated in 2012 and included 16 RCTs (*n* = 1513) in which the 25(OH)D concentration increased by an average of 19.4 nmol/L [40]. Similar to our study, they showed that the 25(OH)D concentration increase is dose-dependent and is affected by the baseline 25(OH)D concentration and country latitude.

Evidence from a long-term population-based Finnish study documented improvement of 25(OH)D concentration after 11 years of fortification. Among non-users of vitD supplements, 25(OH)D concentration increased by 15 nmol/L, and the prevalence of vitD deficiency (< 50 nmol/L) was reduced from 58.5 to 13.7% [64]. Additionally, the study demonstrated the safety of long-term fortifications. In another study among 4-year-old children, vitD intake increased from 176 to 360 IU/day, and the 25(OH)D concentration increased from 54.7 to 64.9 nmol/L after 2 years of implementing the mandatory fortification of milk and margarine [65].

Studies suggest that improving 25(OH)D concentration through food fortification is cost-effective when implemented at the population level [66]. Improving 25(OH)D concentration through population fortification programs in France was estimated to reduce the number of lifetime fractures by 64,932, including 19,500 hip fractures [67]. Comparably, the Canadian Health Measures Survey estimates a reduction in disease incidence, mortality rates, and the total economic burden of diseases such as cancer, cardiovascular disease, dementia, diabetes mellitus, multiple sclerosis, respiratory infections, and musculoskeletal disorders if 25(OH)D concentration is increased to > 100 nmol/L. The estimated reduction in annual economic cost was projected to be 12.5 ± 6.0 billion dollars, and premature deaths were estimated to be reduced by 23,000 (11,000–34,000) [68]. Unfortunately, there is a lack of such cost-effectiveness estimates for pediatrics. Evidence from a pediatric meta-analysis suggested that improved 25(OH)D concentration occurs among children receiving vitD fortified food compared with those receiving supplementation and bolus injection [20].

The utilization of vitD supplementation and compliance with daily intake is a major concern in real-life practice. In Ireland, 17% of pre-school children consume vitD supplements regularly, whereas 77% consume vitD through fortified milk and yogurt [69]. Moreover, vitD supplementation is routinely offered for free in Quebec pharmacies for premature infants. Pharmacy records showed a low utilization of vitD supplements by this vulnerable high-risk group for deficiency [18]. Similarly, a meta-analysis of 18 RCTs on vitD supplementation in adults reported low compliance [70]. Conversely, in our review, compliance was similar among fortified and unfortified food products. Fortification has a major advantage of avoiding issues of affordability, compliance, availability, sustainability, accessibility, acceptability, and knowledge about micronutrient importance, and it does not require mass advertisement [62]. The above reasons possibly explain the marginally higher impact of vitD fortification observed among low-income countries, the improved 25(OH)D concentration among school-aged children compared with children less than 5 years old, and the effectiveness observed with food-fortification strategies adopted by many countries to tackle micronutrient malnutrition [71–76].

In our meta-analysis, the heterogeneity observed with 25(OH)D concentration and the prevalence of vitD deficiency were substantial and similar to those in the previous adult and pediatric meta-analyses [20, 40]. In our study, a combination of characteristics caused the heterogeneity, as illustrated in the meta-regression analysis. Therefore, the resulting treatment effect should be considered with caution. The heterogeneity was partially explained by the utilization of different food vehicles. Among all food vehicles used for fortification, fortified milk compared with control offered the maximum improvement in 25(OH)D concentration even though all other food products were fortified using at least 100 IU/day. This relationship between food type and changes in serum 25(OH)D concentration was not reported in previous RCTs or in population-based cohort studies. This begs the question of possible interactions between vitD and food products that could influence its absorption. Future multi-arm RCTs or network meta-analysis are necessary to provide an accurate estimate. Moreover, the effects of fortifying the milk were more pronounced among children between 5 and 12 years old, likely because the majority of RCTs enrolling children 5–12 years old were performed at schools rather than at clinics, which possibly ensured better compliance and accessibility to the food product.

The impact of fortification was marginally higher in lower-income countries but not to a statistically significant degree. All children included in the trials were adequately nourished, as evidenced by normal BMI values, and they had comparable 25(OH)D concentration at baseline. The presence of policies for vitD fortification in high-income countries has led to the availability of fortified staple food products in the market. This availability may have led to cross-contamination that sustained 25(OH)D concentration in the control group compared with the control groups in the low-income countries where such policies do not exist. Nevertheless, the impact of vitD fortification across different economic statuses was significant despite the lack of statistically differential effects among countries based on their economic status.

At the individual level, an increase in cognitive function by one IQ point is considered small. Specifically, however, methods to improve the cognitive function of children at a societal level are expensive and laborious. The associations between micronutrients and academic performance in school-aged children are not yet well established [77, 78]. However, there is a growing body of evidence linking neurohormonal effects of vitD on the regulation of brain cellular architecture and behavior development [4, 79]. A systematic review of human and animal observational studies observed that low prenatal 25(OH)D concentration led to subtle cognitive and psychological impairments in the offspring [80]. Furthermore, a cross-sectional study suggested a potential association between vitD deficiency during the postnatal period and processing speed and verbal fluency in children [81].

The strengths of our meta-analysis include utilizing sensitive search terms that led to the inclusion of 20 RCTs. Furthermore, 12 RCTs were performed in high- and upper-middle-income countries, and six were performed in lower-middle-income countries, which gives our meta-analysis a global perspective. The inclusion of the high-income countries in the review did not influence the meta-analysis results, as shown in the subgroup analysis. Therefore, these results are generalizable to countries with similar settings. These subgroup analyses and meta-regression can aid policymakers in making informed decisions fitting their own country’s unique population characteristics and needs through utilizing treatment effects from the meta-regression results to arrive to estimated average requirement and recommended dietary allowance values. Nevertheless, meta-regression describes an observational association and should be considered for hypothesis generation not as a proof of causality. Establishing causality for such scenario can be better assessed through individual patient data meta-analysis, which aggregate original research data from each patient involved in trials. Additionally, we reported effect estimates from low RoB RCTs separately to arrive at estimates close to real intervention effects; we also reported the cluster RCTs separately because most cluster RCTs tend to have inflated effect estimates [27, 82]. Although vitD fortification seems to decrease the infection rate and improve cognitive function, which adds further public health gains beyond the direct health benefits of vitD, future research is necessary to confirm these findings, evaluate the cost-effectiveness of fortification for children, evaluate the effects among children with a low BMI status and children with low socioeconomic status, and assess possible adverse events. Moreover, we could not assess IQ confounders because of the small number of studies available for this subgroup analysis.

Preventing vitD deficiency is a public health necessity. VitD micronutrient fortification is an affordable, sustainable, and easily implementable solution for a global public health concern. Implementing vitD food fortification strategies can lead to improved 25(OH)D concentration, reduced vitD deficiency prevalence, and improved children cognitive function. Policymakers across high- and low-income countries are urged to implement mass mandatory vitD fortification strategies of at least one staple food product, preferably fluid milk, and make them widely available, particularly in schools.

## Supplementary information

**Additional file 1.** The search strategy, list of excluded articles, additional summary of included studies

## Data Availability

All data generated or analyzed during this study are included in the published primary articles.

## Abbreviation

VitD: Vitamin D
25(OH)D: 25 hydroxyvitamin D
CI: Confidence interval
SD: Standard deviation
MD: Mean difference
SMD: Standardized mean difference
RR: Relative risk
IQ: Intelligence quotient
RoB: Risk of bias
RCT: Randomized controlled trial
NNT: Number needed to treat
