# Supplementary material for: The impact of vitamin D food fortification and health outcomes in children: a systematic review and meta-regression
Source: Syst Rev. 2020 Jun 16;9:144. doi: 10.1186/s13643-020-01360-3 (PMC7298752; doi:10.1186/s13643-020-01360-3)
Supplement: Supplementary file 1 — Additional file 1. The search strategy, list of excluded articles, additional summary of included studies [file 13643_2020_1360_MOESM1_ESM.docx]

**Search terms:**

**Embase database:**

**Search date: 1974 to May 2019**

| **ID** | **Term** |
| --- | --- |
| 1 | child/ |
| 2 | exp pediatrics/ |
| 3 | child*.mp. |
| 4 | juvenile.mp. |
| 5 | teen*.mp. |
| 6 | young.tw. |
| 7 | adolescen*.mp. |
| 8 | youth.mp. |
| 9 | p??diatric*.mp. |
| 10 | exp vitamin D/ |
| 11 | (vitamin adj (d or d2 or d3)).tw. |
| 12 | vitamin d fortif*.mp. |
| 13 | exp 25 hydroxyvitamin D/ |
| 14 | exp calcifediol/ |
| 15 | exp ergocalciferol/ |
| 16 | exp colecalciferol/ |
| 17 | exp diet supplementation/ |
| 18 | exp trace element/ |
| 19 | exp food/ |
| 20 | fortifi$.mp. |
| 21 | exp fortified food/ |
| 22 | exp food additive/ |
| 23 | random.tw. |
| 24 | placebo.mp. |
| 25 | Double blind.tw. |
| 26 | Single blind.tw. |
| 27 | 1 or 2 or 3 or 4 or 5 or 6 or 7 or 8 or 9 |
| 28 | 10 or 11 or 12 or 13 or 14 or 15 or 16 |
| 29 | 17 or 18 or 19 or 20 or 21 or 22 |
| 30 | 23 or 24 or 25 or 26 |
| 31 | 27 and 28 and 29 and 30 |

**OVID Medline database Epub Ahead of Print, In-Process & Other Non-Indexed Citations, Ovid MEDLINE(R) Daily and Ovid MEDLINE(R)**

**Search date: 1946 to May 2019**

| **ID** | **Term** |
| --- | --- |
| 1 | child/ |
| 2 | exp pediatrics/ |
| 3 | child*.mp. |
| 4 | juvenile.mp. |
| 5 | teen*.mp. |
| 6 | young.tw. |
| 7 | adolescen*.mp. |
| 8 | youth.mp. |
| 9 | p??diatric*.mp. |
| 10 | exp vitamin D/ |
| 11 | (vitamin adj (d or d2 or d3)).tw. |
| 12 | vitamin d fortif*.mp. |
| 13 | exp calcifediol/ |
| 14 | exp ergocalciferol/ |
| 15 | exp Dietary Supplements/ |
| 16 | exp trace element/ |
| 17 | exp food/ |
| 18 | fortifi$.mp. |
| 19 | exp fortified food/ |
| 20 | exp food additive/ |
| 21 | randomized controlled trial.pt. |
| 22 | placebo.mp. |
| 23 | clinical trials as topic.sh. |
| 24 | randomized.ab. |
| 25 | Controlled clinical trial.pt. |
| 26 | randomly.ab. |
| 27 | trial.ti. |
| 28 | 1 or 2 or 3 or 4 or 5 or 6 or 7 or 8 or 9 |
| 29 | 10 or 11 or 12 or 13 or 14 |
| 30 | 15 or 16 or 17 or 18 or 19 or 20 |
| 31 | 21 or 22 or 23 or 25 or 26 or 27 |
| 32 | 28 and 29 and 30 and 31 |

**Global Health database**

**Search date:** 1973 to **May 2019**

| **ID** | **Term** |
| --- | --- |
| 1 | child*.mp. |
| 2 | exp children/ |
| 3 | juvenile.mp. |
| 4 | teen*.mp. |
| 5 | young.tw. |
| 6 | adolescen*.mp. |
| 7 | youth.mp. |
| 8 | p??diatric*.mp. |
| 9 | exp vitamin D/ |
| 10 | (vitamin adj (d or d2 or d3)).tw. |
| 11 | vitamin d fortif*.mp. |
| 12 | vitamin D.sh. |
| 13 | exp ergocalciferol/ |
| 14 | fortification.sh. |
| 15 | fortified food.mp. [mp=abstract, title, original title, broad terms, heading words, identifiers, cabicodes] |
| 16 | Dietary Supplements.mp. [mp=abstract, title, original title, broad terms, heading words, identifiers, cabicodes] |
| 17 | fortifi$.mp. |
| 18 | exp food additive/ |
| 19 | 1 or 2 or 3 or 4 or 5 or 6 or 7 or 8 |
| 20 | 9 or 10 or 11 or 12 or 13 |
| 21 | 14 or 15 or 16 or 17 or 18 |
| 22 | 19 and 20 and 21 |

**Cochrane central database**

**Search date:**: **May 2019**

**ID Search Hits**

#1 child 91197

#2 pediatrics 17410

#3 child* 111985

#4 juvenile 1987

#5 teen* 1756

#6 young 69540

#7 adolescen* 111363

#8 youth 3416

#9 p??diatric* 11615

#10 vitamin D 9064

#11 vitamin d fortif* 397

#12 25 hydroxyvitamin D 1579

#13 calcifediol 371

#14 ergocalciferol 254

#15 colecalciferol 817

#16 diet supplementation 6880

#17 trace element 848

#18 food 26382

#19 fortifi$. 6

#20 fortified food 1619

#21 food additive 288

#22 #1 or #2 or #3 or #4 or #5 or #6 or #7 or #8 or #9 224091

#23 #10 or #11 or #12 or #13 or #14 or #15 9344

#24 #16 or #17 or #18 or #19 or #20 or #21 31612

#25 #22 and #23 and #24 925

# 26 #25in trails 526

**List of excluded articles with reason for exclusion:**

| **Author** | **Year** | **Journal** | **Title** | **Reason for exclusion** |
| --- | --- | --- | --- | --- |
| Abdeen, Z. | 2015 | Public Health Nutrition | Predicted efficacy of the Palestinian wheat flour fortification programme: complementary analysis of biochemical and dietary data | Not fortification |
| Akkermans, M | 2016 | Journal of Pediatric Gastroenterology and Nutrition Conference: 49th Annual Meeting of the European Society for Paediatric Gastroenterology, Hepatology and Nutrition, ESPGHAN | A micronutrient-fortified young child formula improves the iron and vitamin D status of healthy young European children: A randomised double-blind controlled trial | conference paper |
| Al-Khalidi, B | 2015 | Canadian Journal of Dietetic Practice & Research | Bioavailability and Safety of Vitamin D3 from Pizza Baked with Fortified Mozzarella Cheese: A Randomized Controlled Trial | Adult population |
| Al-Ghannami, S. | 2016 | Nutrition | Lipid-soluble nutrient status of healthy Omani school children before and after intervention with oily fish meal or re-esterified triacylglycerol fish oil | not fortification |
| Allen, R. | 2015 | American Journal of Clinical Nutrition | Does fortification of staple foods improve vitamin D intakes and status of groups at risk of deficiency? A United Kingdom modeling study | Wrong study design |
| Andersen, R. | 2014 | British Journal of Nutrition | Dietary effects of introducing school meals based on the New Nordic Diet - A randomised controlled trial in Danish children. The OPUS School Meal Study | Not forttification |
| Ataie-Jafari, A. | 2013 | Clinical Nutrition | A randomized placebo-controlled trial of alphacalcidol on the preservation of beta cell function in children with recent onset type 1 diabetes | Wrong intervention |
| Babu, U. S. | 2010 | American Journal of Clinical Nutrition | Modern India and the vitamin D dilemma: evidence for the need of a national food fortification program. (Special Issue: The vitamin D revolution.) | Adult population |
| Binkley, N. | 2010 | Journal of Clinical Densitometry | Vitamin D fortification of food improves vitamin D status but does not assure adequacy | Adult population |
| Black, L. | 1996 | South African medical journal = Suid-Afrikaanse tydskrif vir geneeskunde | Effect of vitamin-enriched bread on the vitamin status of an isolated rural community--a controlled clinical trial | Wrong study design |
| Bracken, B. | 2014 | FASEB Journal | Cognitive development among preschoolers improves in relation to endline ferritin and vitamin D levels only among recipients randomized to a fortified poultry-based spread (SpammyTM) intervention group | Conference paper |
| Braegger, C. | 2013 | Journal of Pediatric Gastroenterology and Nutrition | Vitamin D in the healthy European paediatric population | Review article |
| Brett, N. | 2016 | American Journal of Clinical Nutrition | Dietary vitamin D dose-response in healthy children 2 to 8 y of age: a 12-wk randomized controlled trial using fortified foods | Wrong comparator |
| Brett, N. | 2016 | FASEB Journal | Lean mass accretion associates with vitamin D intake: A 6 month randomized controlled trial in 2-8 y olds using fortified foods | conference paper |
| Browning, L. | 2014 | Journal of the Science of Food and Agriculture | Vitamin D fortification of eggs for human health | Wrong patient population |
| Calvo, M. | 2004 | American Journal of Clinical Nutrition | Vitamin D fortification in the United States and Canada: current status and data needs | review article |
| Camargo Jr, C. | 2012 | Pediatrics | Randomized trial of vitamin D supplementation and risk of acute respiratory infection in Mongolia | duplicate |
| Carroll, A. | 2014 | Clinical Pediatrics | Vitamin D status in Irish children and adolescents: value of fortification and supplementation | Wrong study design |
| Christofides, A. | 2006 | Maternal & Child Nutrition | Vitamin D: dietary requirements and food fortification as a means of helping achieve adequate vitamin D status | Wrong intervention |
| Cosenza, L. | 2013 | BMC Pediatrics | Calcium and vitamin D intakes in children: a randomized controlled trial | Wrong intervention |
| Cribb, V. | 2015 | Journal of Human Nutrition and Dietetics | Sources of vitamin D and calcium in the diets of preschool children in the UK and the theoretical effect of food fortification | Wrong study design |
| Dong, C. | 2013 | PLoS ONE | Prospective Study on the Effectiveness of Complementary Food Supplements on Improving Status of Elder Infants and Young Children in the Areas Affected by Wenchuan Earthquake | Wrong intervention |
| Ejtahed, H. | 2016 | Journal of the American College of Nutrition | Estimation of vitamin D intake based on a scenario for fortification of dairy products with vitamin D in a Tehranian population, Iran | Wrong patient population |
| Ekbote, V. | 2011 | European Journal of Clinical Nutrition | A pilot randomized controlled trial of oral calcium and vitamin D supplementation using fortified laddoos in underprivileged Indian toddlers | all groups received vitamin d, no control group |
| Flynn, A. | 2009 | Food & Nutrition Research | Intake of selected nutrients from foods, from fortification and from supplements in various European countries | review article |
| Gaffney-Stomberg, E. | 2014 | FASEB Journal | A calcium and vitamin D fortified food product improves bone adaptation during military training | conference paper |
| Ganmaa, D. | 2008 | Asia Pacific Journal of Clinical Nutrition | Effects of vitamin D fortified milk on vitamin D status in Mongolian school age children | Wrong study design |
| Gibson, S. | 2003 | Public Health Nutrition | Micronutrient intakes, micronutrient status and lipid profiles among young people consuming different amounts of breakfast cereals: further analysis of data from the National Diet and Nutrition Survey of Young People aged 4 to 18 years | Wrong study design |
| Gianni, M. | 2014 | BMC Pediatrics | Randomized outcome trial of nutrient-enriched formula and neurodevelopment outcome in preterm infants | Wrong patient population |
| Green, T. | 2010 | Asia Pacific Journal of Clinical Nutrition | Milk fortified with the current adequate intake for vitamin D (5 micro g) increases serum 25-hydroxyvitamin D compared to control milk but is not sufficient to prevent a seasonal decline in young women | Adult population |
| Hernandez, L. | 2014 | FASEB Journal | A tailored, fortified, poultry-based spread (SpammyTM) improves vitamin D and B12 of Guatemalan preschoolers as part of an institutional menu | conference paper |
| Hetzel, A. | 2004 | Cahiers de Nutrition et de Dietetique | Food vectors for vitamin D fortification: simulation of the impact on consumption of target populations | No full text |
| Holick, M. | 1992 | New England Journal of Medicine | The vitamin D content of fortified milk and infant formula | Wrong study design |
| Horn, P. | 2009 | Ugeskrift for laeger | Effect of healthy school meal on selection of blood parameters] | not fortification |
| Hower, J. | 1597 | European Journal of Pediatrics | Vitamin D fortification of growing up milk prevents decrease of serum 25-hydroxyvitamin D concentrations during winter: A clinical intervention study in Germany | duplicate |
| Jacobsen, R. | 2015 | PLoS ONE | Gestational and early infancy exposure to margarine fortified with vitamin D through a National Danish Programme and the risk of type 1 diabetes: the D-tect study | Wrong study design |
| Jensen, C. | 2014 | British Journal of Nutrition | Does prenatal exposure to vitamin D-fortified margarine and milk alter birth weight? A societal experiment | Wrong study design |
| Jensen, C. | 2015 | European Journal of Clinical Nutrition | Prenatal exposure to vitamin-D from fortified margarine and milk and body size at age 7 years | Wrong study design |
| Johnson, J. | 2005 | Journal of Dairy Science | Bioavailability of vitamin D from fortified process cheese and effects on vitamin D status in the elderly | Adult population |
| Keshtkar, A. | 2014 | Osteoporosis International Conference: World Congress on Osteoporosis, Osteoarthritis and Musculoskeletal Diseases, WCO IOF ESCEO | Efficiency of fortified vitamin d milk in adolescents: The community interventional trial (CITFOMI ST) | conference paper |
| Kloosterman, J. | 2007 | European Journal of Nutrition | Safe addition of vitamins and minerals to foods: setting maximum levels for fortification in the Netherlands | Wrong study design |
| Koo, W. | 1995 | Journal of Pediatric Gastroenterology and Nutrition | Effect of three levels of vitamin D intake in preterm infants receiving high mineral-containing milk | Wrong patient population |
| Laaksi, I. T. | 2006 | European Journal of Clinical Nutrition | Vitamin D fortification as public health policy: significant improvement in vitamin D status in young Finnish men | Adult population |
| Lehtonen-Veromaa, M. | 2008 | British Journal of Nutrition | Prospective study on food fortification with vitamin D among adolescent females in Finland: minor effects | Wrong study design |
| Liu, D. S. | 1993 | The American journal of clinical nutrition | Nutritional efficacy of a fortified weaning rusk in a rural area near Beijing | Wrong outcomes |
| Ma, GuanSheng | 2002 | Acta Nutrimenta Sinica | Calcium and vitamin D fortified milk supplementation on bone mineral accretion in pre-pubertal girls in Beijing | Wrong outcomes |
| Ma, GuanSheng | 2006 | Acta Nutrimenta Sinica | Bone mass of middle school girls two years after cessation of calcium-vitamin D-fortified milk supplementation: follow-up study | discontinuation study |
| Mehdi, A. | 1872 | Annals of Nutrition and Metabolism Conference: 20th International Congress of Nutrition Granada Spain Conference Start | Efficacy of a multiple micronutrient fortified milk on cognitive performance of school-age children | conference paper |
| Moore, C. | 2012 | FASEB Journal | Multi-nutrient fortified juices improve vitamin D and vitamin E status in children | Wrong comparator |
| Mukhopadhyay, K. | 2007 | Indian Pediatrics | Effect of human milk fortification in appropriate for gestation and small for gestation preterm babies: a randomized controlled trial | Wrong patient population |
| Neyestani, T. | 2014 | Journal of human nutrition and dietetics : the official journal of the British Dietetic Association | Calcium-vitamin D-fortified milk is as effective on circulating bone biomarkers as fortified juice and supplement but has less acceptance: a randomised controlled school-based trial | duplicate |
| Nissen, J. | 2014 | Genes & nutrition | Real-life use of vitamin D3-fortified bread and milk during a winter season: The effects of CYP2R1 and GC genes on 25-hydroxyvitamin D concentrations in Danish families, the VitmaD study | secondary report of included study |
| Nissen, J. | 2015 | The American journal of clinical nutrition | Common variants in CYP2R1 and GC genes are both determinants of serum 25-hydroxyvitamin D concentrations after UVB irradiation and after consumption of vitamin Dâ‚ƒ-fortified bread and milk during winter in Denmark | Wrong intervention |
| Omidvar, N. | 2012 | Iranian Journal of Nutrition Sciences & Food Technology | Sensory evaluation and assessment of compliance with vitamin D and calcium fortified-milk among school-age children in Tehran | Wrong study design |
| Pietruszka, B. | 2009 | Zywienie Czlowieka i Metabolizm | Vitamin and mineral intake with supplements and fortified foodstuffs by children aged 7-11 years | Wrong study design |
| Piirainen, T. | 2007 | European Journal of Clinical Nutrition | Impact of national fortification of fluid milks and margarines with vitamin D on dietary intake and serum 25-hydroxyvitamin D concentration in 4-year-old children | Wrong study design |
| Sichert-Hellert, W. | 2006 | Journal of Nutrition | Vitamin intakes from supplements and fortified food in German children and adolescents: results from the DONALD Study | Wrong study design |
| Stephen, K. | 1981 | British dental journal | A 4-year double-blind fluoridated school milk study in a vitamin-D deficient area | not vitamin D fortification |
| Zhang, Q. | 2010 | British Journal of Nutrition | The association between dietary protein intake and bone mass accretion in pubertal girls with low calcium intakes | Wrong outcomes |
| Zhang, Q. | 2003 | Zhonghua yu fang yi xue za zhi [Chinese journal of preventive medicine] | Effects of calcium and vitamin D-fortified milk on physical development in school girls aged 10 to 12 years | Wrong study design |
| Zhang, ZheQing | 2014 | Bone | Effects of milk salt supplementation on bone mineral gain in pubertal Chinese adolescents: a 2-year randomized, double-blind, controlled, dose-response trial | Wrong outcomes |
| Zhu, K. | 2008 | Asia Pacific Journal of Clinical Nutrition | Effects of two years' milk supplementation on size-corrected bone mineral density of Chinese girls | Wrong outcomes |
| Zhu, K. | 2005 | The American journal of clinical nutrition | Effects of school milk intervention on cortical bone accretion and indicators relevant to bone metabolism in Chinese girls aged 10-12 y in Beijing | Wrong outcomes |

**S3 Appendix. Additional summary of included studies**

**Akkermans 2017**

| **Methods** | **Study design:** Randomized controlled trial  **Study grouping:** Parallel group |
| --- | --- |
| **Participants** | **Included criteria:** Children aged 12–36 mo with a stable health status (i.e., without any known chronic or recent acute disease.  **Excluded criteria:** being born preterm (32 wk, or,37 wk with a birth weight,1800 g); known infection within the last week or infection needing medical assistance or treatment within the last 2 wk; known hemoglobinopathies; any case of anemia treated within the last 3 mo; a blood transfusion received within the last 6 mo; the presence of a relevant congenital abnormality; chromosomal disorder or severe disease (such as major congenital heart disease or Down syndrome); having a disorder requiring a special diet (such as food intolerance or food allergy or complaints such as reflux, constipation, and cramps); current use of antiregurgitation, antireflux, or laxative medication; participation in any other study involving investigational or marketed products within 2 wk before entering the study; known allergy or intolerance to components of the study products (e.g., milk powder, lactose, orfish protein); and vaccination with a live or live-attenuated vaccine received within the last 2 wk. Finally, parents had to be able to understand the local language and read and fill out questionnaires. |
| **Interventions** | vit d fortification:   - Fortified milk included *vitamin D dose* 68 IU/100ml = ( average of 348 IU per day estimated from the average intake) - *number of patients lost to follow up* : 44(27.8%) - *reasons for lost to follow up* : - (Serious ) adverse event N:6- Withdrawal by subjects N:23- Lost to follow up N:3- Other(not mentioned )N:12   Control   - cow milk - *number of patients lost to follow up* : 47(29.4%) - *reasons for lost to follow up* : - (Serious ) adverse event N:2- Withdrawal by subjects N:22- Lost to follow up N:10- Other(not mentioned )N:13 |

**Economos 2014:**

| Methods | Design: Randomized controlled trial  Group: Parallel group |
| --- | --- |
| Participants | **Inclusion criteria:**   1. Healthy children 2. Aged 6 to 10 years, recruited from the hospital paediatric clinics and through local print and online classified advertisements.   **Exclusion criteria:**   1. History of rickets 2. Diabetes 3. Intestinal malabsorption (ie, cystic fibrosis, fat malabsorption syndrome, or Crohn’s disease) 4. Severe medical illness, including renal failure; allergies to orange juice; any medical conditions precluding daily consumption of orange juice; currently taking, or having taken <1 month before start of study, a prescription vitamin D supplement; and unwillingness to consent/assent to this trial. |
| Interventions | 1. Intervention: received 700 mg calcium and 200 IU vitamin D in 240ml juice  - **Lost to follow up:** 18 - **Number of patients with results:** NA - **Reasons for loss to follow up or incomplete data:** an outlying baseline 25(OH)D level and another due to insufficient baseline data  1. Control: received 700 mg calcium juice.  - **Lost to follow up:** 16 - **Number of patients with results:** NA - **Reasons for loss to follow up or incomplete data:** an outlying baseline 25(OH)D level and another due to insufficient baseline data. |

**Graham 2009:**

| Methods | Design: Cluster randomized controlled trial  Number of clusters: 22  Cluster unit: schools |
| --- | --- |
| Participants | **Inclusion criteria:**   1. primary schools (Years 1–6, or Years 1–8).   **Exclusion criteria:**   1. Not consenting 2. refusal of blood testing 3. Not fasting for blood testing 4. Blood testing was unsuccessful |
| Interventions | Total randomized patients: 172   1. Intervention: 89 randomized receive fortified milk with Vitamin D  - **Lost to follow up:** not mentioned - **Number of patients with results:** not mentioned - **Reasons for loss to follow up or incomplete data:** not mentioned  1. Control 83 randomized receive regular milk.  - **Lost to follow up:** not mentioned - **Number of patients with results:** not mentioned - **Reasons for loss to follow up or incomplete data:** not mentioned - **Compliance:** not mentioned. |
| Outcomes | - Vitamin D level |
|  |  |

**Sun 2011**

| Methods | Design: Randomized controlled trial  Group: Parallel group |
| --- | --- |
| Participants | **Inclusion criteria:** Aged 6 to 8 years of primary school in the first and second grade children.  **Exclusion criteria:**Not clear |
| Interventions | 1. Intervention: 120 randomized receive Vit D fortified 250 ml drink was consumed in the morning every day.  - **Lost to follow up:** not mentioned - **Number of patients with results:** not mentioned - **Reasons for loss to follow up or incomplete data:** not mentioned  1. Control 174 randomized maintained their daily diet without the supplement drinks.  - **Lost to follow up:** not mentioned - **Number of patients with results:** not mentioned - **Reasons for loss to follow up or incomplete data:** not mentioned - **Compliance:** not mentioned. |

**Battiprolu 2006**

| Methods | Design: Cluster randomized controlled trial  Cluster size: 20  Cluster unit: classroom |
| --- | --- |
| Participants | **Inclusion criteria:**   1. 1-6 to 16 y children grades 1 to 10 2. living in residential schools that cater to middle- income, semi-urban populations near Hyderabad, India   **Exclusion criteria:**   1. School having only boys or only girls 2. Orphanages |
| Interventions | 1. Intervention: received 150 ml of base formulation contained wheat flour, malted barley, powdered skim milk, and sugar. The supplement (54 g) provided 7.3 g of protein,2 g of fat, 208 kcal of energy, and 176 mg of calcium. 100% of codex RDA of iron, riboflavin (B2), pyridoxine (B6), vitamin B12, folate, and vitamin C; 50% of the RDA for vitamins A, D, and B1, niacin, calcium, and iodine; and 15% of the RDA for zinc  - **Lost to follow up:** not provided (follow up was reported to be above 80%) - **Number of patients with results:** 110 - **Reasons for loss to follow up or incomplete data:**  not mentioned - **Compliance:** not mentioned  1. Control: received 150 ml of base formulation contained wheat flour, malted barley, powdered skim milk, and sugar. The supplement (54 g) provided 7.3 g of protein,2 g of fat, 208 kcal of energy, and 176 mg of calcium.  - **Lost to follow up:** not provided (follow up was reported to be above 80%) - **Number of patients with results:** 110 - **Reasons for loss to follow up or incomplete data:** not mentioned - **Compliance:** not mentioned |

**Rich-Edwards 2011**

| Methods | Design: Cluster randomized controlled trial  Cluster size: 21  Cluster unit: classroom |
| --- | --- |
| Participants | **Inclusion criteria:** Two public schools were selected for their size, proximity, and comparable sociodemographic profiles.  **Exclusion criteria:** had no known allergies to milk |
| Interventions | 1. Intervention: received 100 IU vitamin D3 per 236 mL milk. Children received 710 mL liquid whole cow milk 7 d/wk of either ultra-high-temperature (UHT)–pasteurized fortified milk shipped from the United States or pasteurized Mongolian milk 2. Control received regular milk.   - The other 2 arms each included children who received a vitamin D3supplement: one arm received 300 IU 7 d/wk, and the other received a total of 13,700 IU in the first 7 d of the trial as a seasonal supplement. Those 2 arms were not included in our analysis. |

**Houghton 2011**

| Methods | Design: Randomized controlled trial  Group: Parallel group |
| --- | --- |
| Participants | **Inclusion criteria:** The inclusion criteria for participation were that the child was ap-parently healthy and 12–20 mo of age inclusive, not currently consuming a fortified milk, and that the caregivers were willing for their child to berandomized to either a meat or a milk intervention.  **Exclusion criteria:** Toddlers were excluded if they had a baseline hemoglobin concentration,105 g/L or a baseline hemoglobin concentration,110 g/L and serum ferritin,12mg/L |
| Interventions | 1. Intervention received 6.0 mg cholecalciferol/100 g milk powder  - **Lost to follow up:** not mentioned - **Number of patients with results:** not mentioned - **Reasons for loss to follow up or incomplete data:** not mentioned  1. Control received red meat.  - **Lost to follow up:** not mentioned - **Number of patients with results:** not mentioned - **Reasons for loss to follow up or incomplete data:** not mentioned - **Compliance:** not mentioned. |

**Powers 2016**

| Methods | Design: Randomized controlled trial, Group: Parallel group |
| --- | --- |
| Participants | **Inclusion criteria:**   1. Female 2. Aged between 16 and19 years at the time of recruitment, who self-reported missing breakfast at least 4 times a week. 3. They had to have no diagnosed illness and have low measures of riboflavin status and haemoglobin, as these have previously been found to track well with self-reported breakfast skipping   **Exclusion criteria:** Potential participants were not recruited if they used multivitamin or iron supplements, had donated blood in the previous 6 months, were pregnant or breast feeding or had an allergy to wheat, barley or milk. |
| Interventions | 1. Intervention received 4.15 μg VitD in 50 g fortified cereal breakfast cereal  - **Lost to follow up:** 5 - **Number of patients with results:** not mentioned - **Reasons for loss to follow up or incomplete data:** one was withdrawn because of iron deficiency anaemia diagnosed by the Royal Hallamshire Hospital Haematology Department.  1. Control received 0.2 μg in 50 g unfortified cereal.  - **Lost to follow up:** not mentioned - **Number of patients with results:** not mentioned - **Reasons for loss to follow up or incomplete data:** not mentioned - **Compliance:** not mentioned. |

**Neyestani 2014**

| Methods | Design: Cluster randomized controlled trial  Cluster size: 6  Cluster unit: schools |
| --- | --- |
| Participants | **Inclusion criteria:**   1. not taking dietary supplements including calcium, vitamin D or omega-3 within the past 3 months prior to the intervention; 2. Not receiving medications that could potentially influence vitamin D metabolism; and 3. Not having any clinical disease that could affect the study results (e.g. allergies,autoimmune disorders, renal, hepatic and endocrinological disorders).   **Exclusion criteria:** unwillingness of either parent or child to participate in the study |
| Interventions | Total randomized patients: 316   1. Cases 146 randomized receive fortified milk containing 100 IU of vitamin D in 200-mL package  - **Lost to follow up:** 8 - **Number of patients with results:** not mentioned - **Reasons for loss to follow up or incomplete data:** The major cause of discontinuation was an unwillingness to donate blood for the second time  1. Control 170 randomized receive regular milk.  - **Lost to follow up:** 5 - **Number of patients with results:** not mentioned - **Reasons for loss to follow up or incomplete data:** The major cause of discontinuation was an unwillingness to donate blood for the second time - **Compliance:** not mentioned. |
| Outcomes | - Vitamin D level - Vitamin D deficiency - Compliance with fortification |
|  |  |

**Madsen 2013**

| Methods | Design: Randomized controlled trial, Group: Parallel group |
| --- | --- |
| Participants | **Inclusion criteria:**  1- Age between 4 and 60 y  2- Permanent address in the Gladsaxe Municipality  **Exclusion criteria:**  1- Pregnancy  2- Disease or use of a medication that would influence vi- tamin D metabolism (including dietary supplements with .10 mg vitamin D/d for children and .5 mg vitamin D/d for adults, which corresponded to the typical amounts in multivitamin supplements in Denmark) |
| Interventions | 1. Intervention: received bread and milk: milks was organic homogenized 0.5% fat milk produced by Arla Foods A/S. bread was white bread, brown bread, rye bread, and buns that were baked from wheat- and rye-flour blends.  - **Lost to follow up:** 36 - **Reasons for loss to follow up or incomplete data:** Withdrew before start:   did not want to provide blood sample: (8 children), did not find the time for blood sample: (3)  Withdraw during study: Traveling: (6), Lost contact: (8), did not want to provide the last blood sample: (7 children),  Excluded from analysis: Insufficient blood sample: (4)  2. Control received regular bread and milk.   - **Lost to follow up:** 20 - **Number of patients with results:** 48 - **Reasons for loss to follow up or incomplete data:** did not want to provide blood, private reason and insufficient blood collection. |

**Kuriyan 2016**

| Methods | Design: Randomized controlled trial  Group: Parallel group |
| --- | --- |
| Participants | **Inclusion criteria:**   1. Normal healthy children 2. Age range of 7-10 years.   **Exclusion criteria:**  1- girls who had attained menarche  2- presence of severe anemia (Hb < 8 g/dl)  3- moderately/severely undernourished children (BMI for age z-score < -2SD, WHO)  4- organ failure (as assessed by medical history)  5- physical disability  6- recent history of serious infections  7- food allergies or intolerance  8- children consuming nutritional supplements and or health food drinks  9- participating in any nutritional study in the previous year  10- child’s family likely to move out of the study area within the period of study |
| Interventions | 1. Intervention: receive multi micronutrient fortified malt and cocoa based milk 2.9 mcg Per 2 servings, twice daily, 6 days a week for a period of 5 months.  - **Lost to follow up:** 3 - **Number of patients with results:** not mentioned - **Reasons for loss to follow up or incomplete data:** taking medication, left school, not presenting for long time  1. Control receive un-fortified malt and cocoa based milk 0.4 mcg Per 2 servings of placebo twice daily, 6 days a week for a period of 5 months..  - **Lost to follow up:** 0 - **Number of patients with results:** not mentioned - **Reasons for loss to follow up or incomplete data:** NA |

**Khadgawat 2013**

| Methods | Design: Randomized controlled trial  Group: Parallel group |
| --- | --- |
| Participants | **Inclusion criteria:**  Healthy children aged 10–14 years (boys—332; girls—464) from three paying schools, located in the National Capital Region of Delhi  **Exclusion criteria:**  Subjects with diagnosed diabetes mellitus or past/current history of renal stones, and any known systemic, endocrine or metabolic disorder. Similarly, subjects receiving any medications known to interact with vitamin D metabolism (steroids, thiazide diuretics, phenytoin, phenobarbitone, and antitubercular drugs) or taking or had taken vitamin D supplementation in last 6 months, were also excluded. Additional exclusion criteria applied were: subjects with hypercalcemia, hypercalciuria, and S.25(OH)D level more than 100 ng/ml |
| Interventions | 1. Intervention receive 200 ml of milk fortified with either 600 and 1,000 IU of vitamin per day respectively.  - **Lost to follow up:** 45 - **Number of patients with results:** not mentioned - **Reasons for loss to follow up or incomplete data:**   1-did not turn up for repeat sampling at the completion of the study  2-children left schools as their parents moved out of Delhi)   - **Compliance:** not mentioned  1. Control receive unfortified milk  - **Lost to follow up:** not mentioned - **Number of patients with results:** not mentioned - **Reasons for loss to follow up or incomplete data:** NA |

**Hower 2013**

| Methods | Design: Randomized controlled trial  Group: Parallel group |
| --- | --- |
| Participants | **Inclusion criteria:**  Children aged 2–6 years Healthy, who provided Consent.  **Exclusion criteria:**  Children with hyperglycemia, 25(OH)D concentrations below10 ng/mL2 and above 100 ng/mL, intolerance to cow's milk, concomitant intake of vitamin D or other vitamin preparations, and children participating in another clinical trial were excluded from the study. |
| Interventions | 1. Intervention receive fortified milk 2.85 mcg/100ml (112 IU) 350 ml per day  - **Lost to follow up:** 16 - **Number of patients with results:** not mentioned - **Reasons for loss to follow up or incomplete data:**   Low vit D, milk intolerance, milk not accepted anymore, lost follow up.   - **Compliance:** not mentioned  1. Control receive semi-Skimmed milk vitamin D (0.03 μg/100 mL) 350 ml per day  - **Lost to follow up:** 23 - **Number of patients with results:** not mentioned - **Reasons for loss to follow up or incomplete data:** Low vit D, milk intolerance, milk not accepted anymore, lost follow up. |

**Hettiarachchi 2010**

| Methods | Design: Randomized controlled trial  Group: Parallel group |
| --- | --- |
| Participants | **Inclusion criteria:**  Healthy children aged 3-5 years (an equal proportion of male and female), who were free of infectious diseases or diarrhea within the preceding two weeks were selected from the University field training area of the Faculty of Medicine, Galle, Sri Lank. all children were pre-treated with anti-parasitic agent before he study  **Exclusion criteria:**  Subjects, who had a history of medical conditions lasting more than one month, consumed medications, including vitamin or mineral preparations for more than one month, were not included in the study. Children with height or weight below the 3rd centile were also exclude. |
| Interventions | Intervention: receive thriposha (Cereal fortified with Vit D and other micronutrient) 200 IU in 100g of thriposha   - **Lost to follow up:** None   Control receive thriposha (Cereal not fortified with Vit D and other micronutrient)   - **Lost to follow up:** None |

**Du 2004**

| Methods | Design: Cluster randomized controlled trial  Cluster size: 9  Cluster unit: schools |
| --- | --- |
| Participants | **Inclusion criteria:** Adolescent, free of any disease might affect bone health.  **Exclusion criteria:** Non healthy teenagers . |
| Interventions | 1. Intervention receive milk fortified with calcium and vit D 144 ml intervention milk (245 mg Ca and 3.33 mg cholecalciferol)  - **Lost to follow up:** 18 - **Reasons for loss to follow up or incomplete data:** non-compliance, fear of venipuncture and changing school  1. Control receive unfortified milk  - **Lost to follow up:** 29 - **Reasons for loss to follow up or incomplete data:** fear of venipuncture and changing school |

**Benjeddou 2019**

| **Methods** | **Study design:** Randomized controlled trial  **Study grouping:** Parallel group |
| --- | --- |
| **Participants** | **Inclusion criteria:**  Healthy children with no health problems such as anemia or chronicdiseases. In addition, the participants were not allowed to take any other food supplements before and during the period of the intervention.  **Excluded criteria:** Chronic disease, or on any supplement |
| **Interventions** | Intervention group received 200ml of Milk daily containing 3ng vitD  **Lost to follow up:** 16  ***Reasons for lost to follow up*:** Refused to give blood (n=5) Absent on the day of blood sampling (n=4 ) Change of school (n=1)  Control received 200 ml milk daily containing <1.5 ng vitD  **Lost to follow up:** 23  *reasons for lost to follow up* : Refused to give blood (n=8) Absent on the day of samples withdrawal (n=5) Change of study area (n=2) lood sampling |
| **Outcomes** | *vitamin D level*  *vitamin D deficiency prevalence* |

**Brett 2018**

| **Methods** | **Study design:** Randomized controlled trial  **Study grouping:** Parallel group |
| --- | --- |
| **Participants** | **Inclusion criteria:** 2–8 y of age, consuming milk products regularly, within± 2BMI z scores from 0 for sex and age based on WHO growth charts or body fat percentage within normal ranges, and not taking supplements containing vitamin D  **Exclusion criteria:** chronic diseases or medications known to affect vitamin D, known anemia, small size at birth, or preterm birth at <37 wk of gestation |
| **Interventions** | Intervention:  33 g Cheddar cheese /day or two 93 ml-drinkable yogurts/day. The cheese contained 300 IU vitamin D3/33 g and yogurt beverages contained 150 IU/93 mL  Control:  33 g cheddar cheese/d or two 93-mL drinkable yogurts/d. included vitD 140–195 IU/d   - **Lost to follow up:** 2 - **Reasons for loss to follow up or incomplete data:** 1 loss follow up after first visit and the other one did not like study product |
| **Outcomes** | - Vit D concentration - Vit D deficiency |

**Ohlund 2017**

| **Methods** | **Study design:** Randomized controlled trial  **Study grouping:** Parallel group |
| --- | --- |
| **Participants** | **Inclusion criteria:** children living in northern and southern Sweden were included. Healthy 5- to 7-y-old children 50% with fair and 50% with dark skin.  **Exclusion criteria:** Vitamin D supplement |
| **Interventions** | **Intervention:** 200 ml of milk per day, first group containing 10ng\day second group 25 ng\day vitD   - **Lost to follow up:** 12   **Control:**  200 ml of milk per day containing 2 mcg\day vitD   - **Lost to follow up:** 5   **Reasons for loss to follow up or incomplete data:**  Problems with accepting the taste of the study product and, in a few cases, problems with blood sampling, and 1 child could not travel with the study product when going abroad. |
| **Outcomes** | - Vit D concentration - Vit D deficiency - Compliance |
